# Mitotic Disruption and Cytoskeletal Alterations Induced by *Acorus calamus* Essential Oil: Implications for Bioherbicidal Potential

**DOI:** 10.3390/ijms26188933

**Published:** 2025-09-13

**Authors:** Mateusz Wróblewski, Natalia Gocek, Aneta Żabka, Justyna T. Polit

**Affiliations:** 1 Department of Cytophysiology, Faculty of Biology and Environmental Protection, University of Lodz, 90-236 Lodz, Poland; mateusz.wroblewski@biol.uni.lodz.pl (M.W.); natalia.gocek@biol.uni.lodz.pl (N.G.); aneta.zabka@biol.uni.lodz.pl (A.Ż.); 2 Doctoral School of Exact and Natural Sciences, University of Lodz, 90-237 Lodz, Poland

**Keywords:** phytotoxicity, mitotic index, chromosomal aberrations, CDKA, epigenetics, cytoskeleton

## Abstract

Essential oils are increasingly recognized as promising agents for sustainable weed control due to their selectivity and complex modes of action. This study evaluated the effects of *Acorus calamus* essential oil (SEO) on mitosis in two Fabaceae species (*Vicia faba*, *Lupinus luteus*) and two Brassicaceae species (*Brassica napus*, *Arabidopsis thaliana*) treated with species-specific IC_50_ concentrations (0.03%, 0.025%, 0.01%, and 0.005%, respectively). Previous research showed that SEO induces oxidative stress and S-phase delay via genome instability. Here, SEO consistently disrupted mitosis across all species, reducing mitotic index by 50–60%, decreasing Cdc2 (CDKA homolog) levels, and causing chromosomal aberrations, including uneven chromatin condensation, sticky chromosomes, bridges, and micronuclei. Cells accumulated in metaphase and exhibited abnormal karyokinetic and cytokinetic spindles. Immunolabeling revealed thick, tightly packed microtubules and actin filaments, indicating excessive stabilization and impaired reorganization. Epigenetic regulation was also affected: H3T3 phosphorylation was abnormally strong, widely distributed, and persistent into anaphase/telophase, while H3S10Ph intensity was weakened. These results suggest that SEO targets multiple components of mitotic machinery and epigenetic control, regardless of species. The observed selectivity depends on dosage, not mechanism. This multi-targeted action may limit the development of plant resistance, supporting the potential of SEO as a bioherbicide in sustainable agriculture.

## 1. Introduction

The extensive use of chemical pesticides, driven by global demand for high crop yields and aesthetic products, has significant detrimental effects on the environment and human health. Due to their poor biodegradability, crops and soil are abundant in their toxic remains. Nowadays, increasing efforts are being undertaken to reduce the use of chemical pesticides, which induce resistance in target organisms and hamper the well-being of non-targeted ones [1,2,3]. Unfortunately, the sophistication of the development and testing process of new, safer substances and the long legislation procedures in light of systematic pesticide withdrawal from the market cast a shadow on efficient crop production. The ambitious idea of replacing or even reducing the use of synthetic pesticides with biologically originated compounds in a short time is a global challenge [4]. Despite the serious problems, such as the intensification of pest metabolism due to global warming, weed infestation causes enormous crop losses exceeding twice those caused by pests [5]. Weeds compete with crop plants for shared resources such as light, water, and nutrients, which depletion is detrimental for crops, especially during the initial growth stages. Recent studies indicate that weeds are responsible for up to 50% of crop losses even when the best practices of weed management are applied [6]. Moreover, in slow-growing legumes, crop losses are said to be up to even 97% [7].

Some crop plants can control the weed competitors by excreting chemical substances that affect the growth of the neighboring plants. The phenomenon called allelopathy is based on a chemical interaction between two plant species where one excretes allelochemicals represented by plant secondary metabolites, which are detrimental to the second one. Allelopathy has been observed for many plant species [8,9,10,11,12,13]. In recent years, increasing attention has been paid to allelochemicals of the benzoxazinoid family produced by rye, wheat, and maize [14]. Allelopathy is then a promising strategy in modern, environmentally friendly crop protection from weeds.

Essential oils (EOs) are other allelochemicals originating from plant secondary metabolism. These volatile substances exhibiting allelopathic activities are complex mixtures of 20–60 active ingredients classified as terpenes, phenylpropanoids, alcohols, aldehydes, and esters [15,16]. They have attracted considerable attention due to their diverse bioactive properties [8,17,18,19,20,21,22]. Beyond general phytotoxic effects, these metabolite blends can also interfere with fundamental cellular functions [10,11,21,23,24,25,26,27]. At the organismal level, EOs commonly inhibit seed germination, root and shoot elongation, and leaf development, as shown, inter alia, for *Citrus aurantiifolia* [28] and *Mentha longifolia* EO [29] and for many other EOs widely described by Verdeguer et al. [16]. These morphological effects result from disruptions of fundamental physiological processes, including photosynthesis, mitochondrial respiration (e.g., by *Origanum vulgare* EO [23] and cinnamaldehyde in cinnamon oil [24]), and hormonal regulation (e.g., by *Artemisia argyi* extract [30]) At the cellular level, EOs induce oxidative stress through excessive production of reactive oxygen species (ROS), lipid peroxidation, and destabilization of membranes and ion balance, as reported for lemongrass, cinnamon, peppermint, well as for individual compounds such as carvacrol and eugenol. Terpene-rich EOs also impair plant growth by inhibiting mitosis, leading to reduced DNA synthesis, a lower mitotic index, phase-specific arrest, and chromosomal aberrations caused by cytoskeletal dysfunctions [10,16,17,20,23,25,31,32]. For instance, *Schinus terebinthifolius* and *Schinus molle* EOs interfere with the cell cycle by disrupting chromatin condensation and spindle organization during mitosis [33]. Taken together, these multi-level phytotoxic effects highlight the potential of EOs as promising candidates for environmentally safe bioherbicide formulations in sustainable agriculture.

An essential oil possessing intriguing properties is produced in rhizomes of sweet flag (*Acorus calamus* L.) [34,35,36]. It consists of different classes of chemical substances, where phenylpropanoids, including α-, β-, and γ-asarones, are the most abundant. Numerous studies have confirmed the medicinal properties of the sweet flag essential oil (SEO), which has a long history of use in traditional Chinese medicine [35,36,37,38]. However, asarones, which are abundantly present in the oil, can exhibit either pro- or antioxidative activity depending on the concentration [39]. This dualistic mode of action has become a subject of scientific debate, particularly in light of its potential genotoxic and cytotoxic effects, which remain inconclusive due to insufficient data [40]. Studies on the impact of SEO on plant growth remain limited. However, available data indicate its selective inhibitory effects on seed germination and early seedling development, highlighting it as one of the most potent EOs in this context [12,22]. At the cellular level, SEO at its IC_50_ concentration (causing a 50% reduction in root growth), provokes oxidative stress characterized by excessive accumulation of ROS, enhanced lipid peroxidation, and the induction of DNA double-strand breaks. This leads to replication stress, manifested by reduced proportion of cells entering S phase, and impaired progression through DNA synthesis, primarily due to diminished replication dynamics within condensed heterochromatin. Notably, despite these genotoxic effects, SEO does not trigger endoreduplication, apoptotic DNA fragmentation, or extensive cell death [22,41]. Notably, SEO influence on cell cycle regulation in meristematic tissues is of particular interest, as these regions play a critical role in plant growth and development [42,43]. Within the cell cycle, multiple potential targets may be affected by EO constituents, including molecular regulators of cell cycle progression, the integrity of the dynamic cytoskeleton, and structural remodeling of chromatin [16]. Gaining insight into the mechanisms by which substances contained in SEO affect cell proliferation is essential for evaluating both their biological relevance and potential applications in crop management and plant protection.

Plant cell proliferation is highly sensitive to environmental factors and relies on conserved eukaryotic mechanisms with plant-specific features, regulated mainly at the G1/S and G2/M transitions [44,45,46,47,48,49]. Progression through the cycle depends on cyclin-dependent kinase/cyclin complexes (CDK/Cyc) [50,51,52,53,54,55], whose activity is modulated by CDK-activating kinases (CAKs), CDK/Cyc inhibitors (CKIs) [53,56,57,58,59,60,61,62,63], antagonistic regulators such as WEE1 kinase and CDC25-like phosphatase [64,65,66], and other protein phosphatases (PP1/PP2A). The RBR/E2F/DP pathway also plays a central role, linking cell cycle control with transcription of S-phase genes [67,68,69].

In early G1, CDKA/CycD complexes, once activated by CAK and released from CKI or WEE1 inhibition, phosphorylate RBR proteins, thereby releasing E2F/DP to induce transcription required for DNA replication. Replication origin licensing and checkpoints mediated by ATM/ATR kinases and CHK-like proteins then secure accurate initiation and fidelity of S phase [52,67,70,71,72,73].

During G2, increasing levels of CycA, B, and D form complexes with CDKA (the plant homolog of yeast and animal Cdc2/CDK1) or with the plant-specific CDKB to promote mitotic entry [45,66,67,68,69]. These complexes are suppressed by CKIs or by WEE1-mediated phosphorylation and activated by CAK [74,75,76]. A CDC25-like phosphatase may also remove inhibitory phosphates, although in higher plants only a truncated homolog has been identified, retaining the catalytic but lacking the regulatory domain, suggesting a plant-specific mechanism of G2/M regulation. CDC25 activity itself is antagonized by PP1 and PP2A [69,77].

Active CDK/Cyc complexes further phosphorylate CKIs, preventing secondary repression, and act on substrates that drive mitosis. These include transcription factors such as MYB3R, which activate genes encoding mitotic proteins (e.g., CDC20, CycA, CycB, NACK1) [54,78], as well as structural components: lamins (nuclear envelope breakdown), histone H1 (chromosome condensation), and nucleolin (nucleolar disorganization) [79,80,81]. These molecular reactions control the distribution of the replicated DNA between daughter cells, with the participation of dynamic cytoskeletal rearrangements [82,83].

In plant cells, early mitotic events are coordinated by the preprophase band (PPB), a transient microtubule–actin structure that marks the future division plane [83]. Although it disassembles at prophase, its positional information is retained through cortical landmarks and cytoskeletal reorganization, ensuring correct spindle orientation and division plane placement. During prophase, microtubules around the nuclear envelope reorganize into a bipolar spindle, which in plants forms without centrosomes; instead, microtubule-rich regions at opposite nuclear sides act as poles interconnected by microtubule-associated proteins (MAPs) and motor proteins [83]. Actin filaments form a supportive cage around the spindle, maintaining its orientation and stability [84,85,86]. After nuclear envelope breakdown, microtubules rapidly polymerize and attach to kinetochores, forming stable kinetochore fibers reinforced by γ-tubulin ring complex (γ-TuRC). In metaphase, augmin-dependent γ-TuRC recruitment drives branching nucleation, enhancing spindle robustness and chromosome alignment [83]. During anaphase, chromatids separate through coordinated microtubule depolymerization at both plus and minus ends, while central spindle microtubules are continuously regenerated and stabilized. Kinesin-14 motors generate sliding forces that maintain spindle integrity and elongation [83]. Finally, in telophase, cytokinesis is initiated by phragmoplast formation, a dynamic structure of antiparallel microtubules, actin filaments, and associated proteins that direct vesicles with cell wall precursors to the division site, facilitating cell plate assembly and the formation of the new cell wall. MAP65 proteins cross-link antiparallel microtubules, maintaining phragmoplast integrity and guiding its expansion until the cell plate fuses with the parental plasma membrane [83,86].

Alongside cytoskeletal reorganization, chromatin undergoes major structural changes during mitosis. In the G2/M transition, previously decondensed and transcriptionally active chromatin compacts into mitotic chromosomes through the action of structural maintenance of chromosome (SMC) complexes: condensins drive condensation, while cohesins hold sister chromatids together until anaphase. Together, they provide the scaffold required for chromosome architecture and accurate segregation [87]. An early epigenetic event in mitotic chromatin is histone H3 phosphorylation at threonine 3 (H3T3Ph) by Haspin kinases. In plants, H3T3Ph is broadly distributed and promotes chromatin condensation and CPC (chromosome passenger complex) recruitment. CPC, which accumulates at kinetochores before nuclear envelope breakdown, ensures centromeric localization of Aurora B kinase, a key mitotic regulator required mainly for division initiation [84,88,89]. Once recruited, Aurora B phosphorylates histone H3 at serine 10 (H3S10Ph), a conserved modification appearing in late G2 within pericentromeric heterochromatin and disappearing in telophase [89,90]. H3S10Ph supports condensation and facilitates cohesin release from chromosome arms, promoting sister chromatid resolution. Proper centromeric positioning of Aurora B stabilizes kinetochore–microtubule attachments, which is essential for accurate chromosome segregation [88,89].

As in other eukaryotes, plant mitosis is safeguarded by the spindle assembly checkpoint (SAC), which delays anaphase until all kinetochores are properly attached to spindle microtubules, thereby ensuring accurate chromosome segregation and genome stability [83]. Erroneous attachments activate the SAC and trigger formation of the mitotic checkpoint complex (MCC), consisting of MAD2, BMF3, and CDC20. In this complex, CDC20 is sequestered and unable to activate the anaphase-promoting complex/cyclosome (APC/C), an E3 ubiquitin ligase required for separase activation and chromatid separation. SAC also cooperates with the chromosome passenger complex (CPC) to correct improper kinetochore–microtubule interactions, ensuring that APC/C activation occurs only when all chromosomes are correctly aligned under bipolar tension [88]. This multilayered regulation highlights the essential role of SAC in error-free mitotic progression.

This study aimed to elucidate the bioherbicidal potential of SEO by examining its effects on mitosis, the final stage of the plant cell cycle. Despite documented bioactivity in animal models and *in vitro* systems, the cellular and molecular mechanisms of SEO in plants remain poorly understood, highlighting the novelty of this research [39,42,91]. Using a model previously applied to S-phase studies, we investigated four plant species selected to represent both crops and weeds. The Fabaceae crops *Vicia faba* L. var. *minor* and *Lupinus luteus* L., characterized by large seeds and slow early growth, are particularly susceptible to weed competition and limited selective herbicides [92,93]. The Brassicaceae species *Brassica napus* L. and *Arabidopsis thaliana* (L.) Heynh were chosen as representatives of undesirable plants in crop systems, while *B. napus* volunteer seedlings can threaten legume production. *A. thaliana* serves both as a common weed and a well-established model organism for future molecular studies [94]. Species-specific IC_50_ values of SEO were determined to allow for comparisons under equivalent stress conditions [22,41].

We hypothesized that SEO disrupts mitosis by altering the abundance of key cell cycle regulator Cdc2 (the conserved homolog of plant CDKA), spindle organization, and mitotic chromatin structure, and that these effects may vary among species, revealing taxon-specific responses or family-level patterns. To test this, we assessed the abundance of Cdc2, mitotic indices, aberrations in mitotic figures, spindle microtubule and actin filament organization, and histone phosphorylation at H3T3 and H3S10. This framework provides both a mechanistic understanding of SEO action in plant cells and insights into its potential as a selective, eco-friendly bioherbicide.

## 2. Results

### 2.1. Effects of SEO on Mitotic Activity and Chromosomal Integrity in Root Meristematic Cells

**Figure 1:**
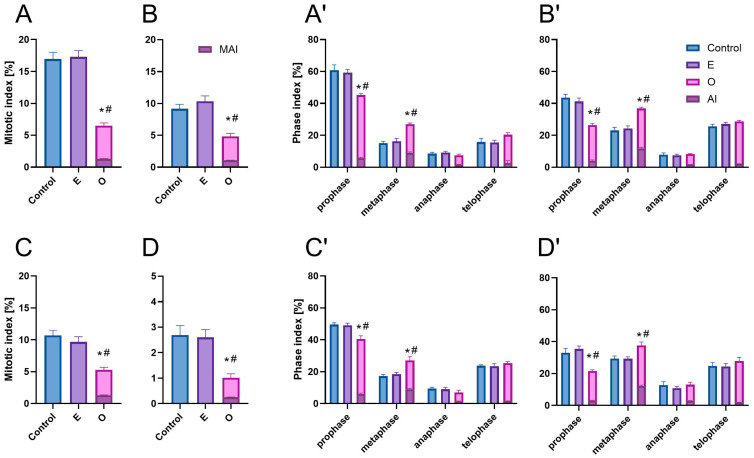
Mitotic indices in root meristem cells of (**A**) *V. faba*, (**B**) *L. luteus*, (**C**) *B. napus*, (**D**) *A. thaliana*, and phase indices (**A′**–**D′**, respectively), including mitotic aberration indices—MAI and phase aberration indices—AI, after 24 h seedling incubation in water—Control, emulsifier solution—E, or emulsified SEO at the IC_50_ concentration—O. Data are presented as mean indices ± SEM from three biological replicates. Statistical differences were assessed using Student’s *t*-test at *p* ≤ 0.05. Asterisk (*) and hash (#) marks denote significant differences compared to the control and the emulsifier, respectively.

Mitosis represents the shortest phase of the cell cycle, and cell divisions in the root meristem occur asynchronously and with varying intensity among plant species. Consequently, in control root meristems, the mitotic index (MI) was relatively low and species-specific, averaging approximately 17% in *V. faba*, 9% in *L. luteus*, 11% in *B. napus*, and 3% in *A. thaliana* (Figure 1A–D). The predominance of early mitotic stages among dividing cells reflects the inherently extended duration of prophase, which is the longest phase of mitosis. Accordingly, prophase and metaphase accounted for 61% and 15% of mitotic figures in *V. faba*, 44% and 23% in *L. luteus*, 50% and 17% in *B. napus*, and 33% and 29% in *A. thaliana*, respectively (Figure 1(A′–D′)). Therefore, the later mitotic stages (anaphase and telophase) collectively constituted a smaller proportion: 24% in *V. faba*, 33% in *L. luteus*, 33% in *B. napus*, and 38% in *A. thaliana* (Figure 1(A′–D′)).

The emulsifier, used to prepare a uniformly distributed SEO emulsion in the culture medium where the seedlings were grown, had no statistically significant effect on the mitotic index (Figure 1A–D) or on the distribution of mitotic phases (Figure 1(A′–D′)). Mitotic figures in both control and emulsifier-treated root meristems displayed proper morphology.

**Figure 2:**
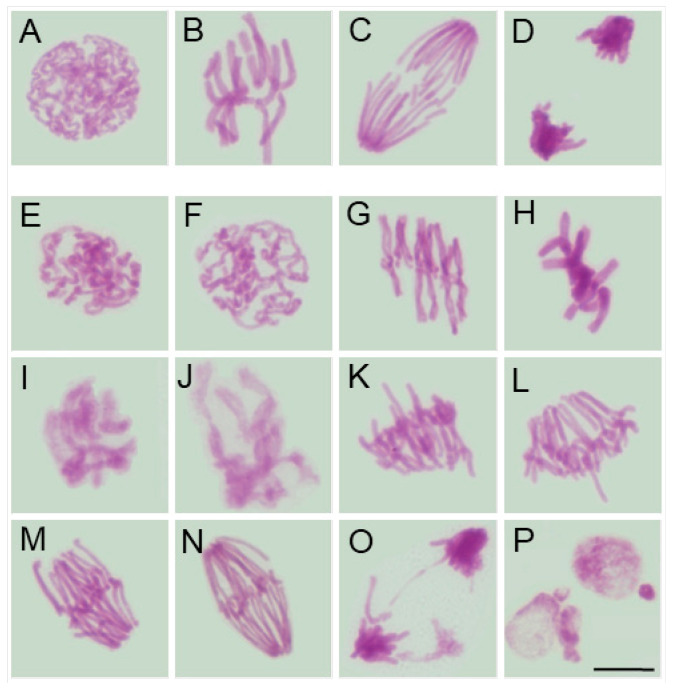
Mitotic figures in root meristem cells of *V. faba* after 24 h seedling incubation in water—Control (prophase: (**A**); metaphase: (**B**); anaphase: (**C**); telophase: (**D**)) and in emulsified SEO at the IC_50_ concentration (prophase with uneven chromosome condensation and Feulgen-positive foci: (**E**,**F**); metaphase with some telomere clustering: (**G**); metaphase with excessively condensed chromosomes: (**H**); metaphase with insufficiently condensed chromosomes: (**I**,**J**); early and late anaphase with sticky telomeric ends: (**K**,**L**) and (**M**,**N**) telophase with chromosomal bridges: (**O**); post telophase with micronuclei: (**P**)). Scale bar: 10 µm.

**Figure 3:**
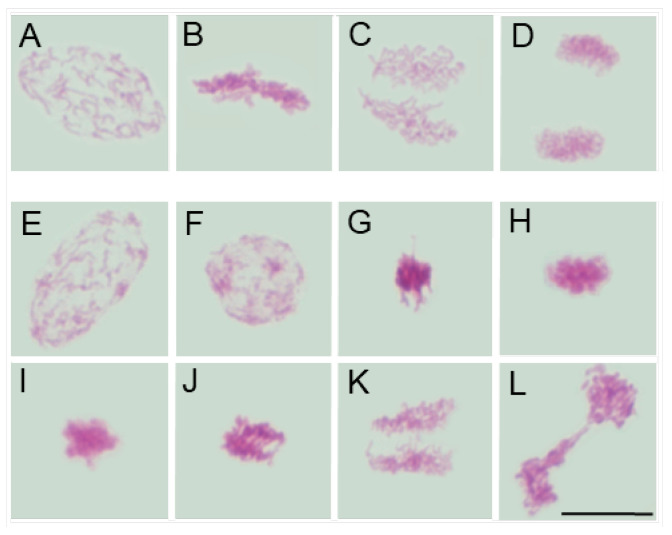
Mitotic figures in root meristem cells of *L. luteus* after 24 h seedling incubation in water—Control (prophase: (**A**); metaphase: (**B**); anaphase: (**C**); telophase: (**D**)) and in emulsified SEO at the IC_50_ concentration (prophase with uneven chromosome condensation and Feulgen-positive foci: (**E**,**F**); metaphase with excessively condensed chromosomes: (**G**); metaphase with insufficiently condensed chromosomes: (**H**); decondensed and condensed anaphase with sticky telomeric ends: (**I**,**J**); early and late telophase with chromosomal bridges: (**K**,**L**)). Scale bar: 10 µm.

**Figure 4:**
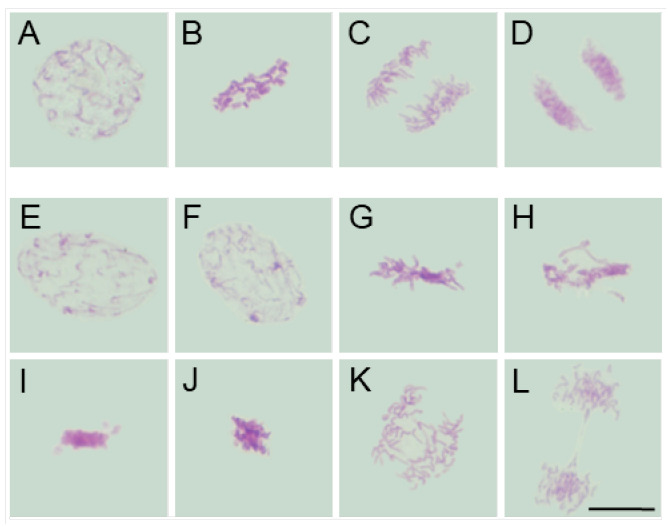
Mitotic figures in root meristem cells of *B. napus* after 24 h seedling incubation in water—Control (prophase: (**A**); metaphase: (**B**); anaphase: (**C**); telophase: (**D**)) and in emulsified SEO at the IC_50_ concentration (prophase with uneven chromosome condensation and Feulgen-positive foci: (**E**,**F**); metaphase with excessively condensed, misaligned and scattered chromosomes: (**G**,**H**); metaphase with insufficiently condensed chromosomes: (**I**); condensed anaphase with sticky telomeric ends: (**J**); anaphase with sticky telomeric ends and chromosomal bridges: (**K**); telophase with a chromosomal bridge and atypical chromatin condensation: (**L**)). Scale bar: 5 µm.

**Figure 5:**
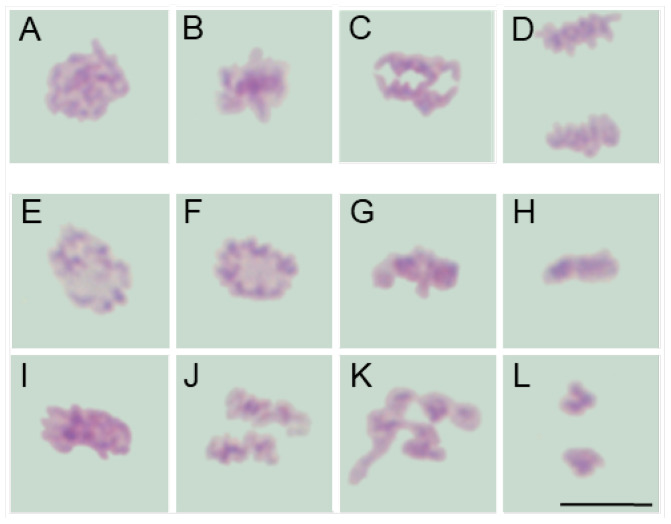
Mitotic figures in root meristem cells of *A. thaliana* after 24 h seedling incubation in water—Control (prophase: (**A**); metaphase: (**B**); anaphase: (**C**); telophase: (**D**)) and in emulsified SEO at the IC_50_ concentration (prophase with uneven chromosome condensation and Feulgen-positive foci: (**E**,**F**); metaphase with excessively condensed chromosomes: (**G**); metaphase with insufficiently condensed chromosomes: (**H**); anaphase with sticky telomeric ends: (**I**); anaphase with uneven chromosome condensation: (**J**); anaphase with chromosomal bridges: (**K**); telophase with atypical chromatin condensation: (**L**)). Scale bar: 5 µm.

Despite interspecific differences in chromosome number, size, and morphology (compare Figure 2A–D, Figure 3A–D, Figure 4A–D and Figure 5A–D), chromosomes in all four species appeared structurally normal, without signs of breakage, uneven chromatin condensation, or under-replicated chromatid fragments. They aligned correctly on the metaphase plate and segregated faithfully, forming two genetically balanced daughter nuclei, as previously demonstrated in the analysis of interphase nuclei [41].

Following exposure to SEO, notable alterations were observed in the mitotic parameters of the root meristems. The mitotic index significantly declined to an average of 6.5% in *V. faba* (38% of control), 5% in *L. luteus* (just over 50% of control), 5% in *B. napus* (just under 50% of control), and 1% in *A. thaliana* (33% of control) (Figure 1A–D). The distribution of cells across mitotic phases also changed, particularly within early mitosis. All species exhibited a reduction in the proportion of prophase cells (by 15 and 17% in Fabaceae or 9 and 12% in Brassicaceae), accompanied by an increase in metaphase cells (by 12 and 14% in Fabaceae or 10 and 8% in Brassicaceae) (Figure 1(A′–D′)).

Moreover, in SEO-treated root meristems of all four species, alongside mitotic figures displaying a normal appearance, cells were also observed to exhibit similar chromosomal structural abnormalities at consecutive stages of mitosis. In prophase, chromatin displayed an uneven degree of condensation, with clearly demarcated, intensely Feulgen-positive regions, likely corresponding to heterochromatic domains (compare figures: Figure 2E,F, Figure 3E,F, Figure 4E,F and Figure 5E,F vs. Figure 2, Figure 3, Figure 4 and Figure 5A). Metaphase abnormalities were diverse in nature. One such disturbance involved telomere clustering, most clearly observed in *V. faba* due to its large chromosomes (Figure 2G), or misaligned and scattered chromosomes at the metaphase plate, as seen in *B. napus* (Figure 4G). Other anomalies included excessively condensed chromosomes densely packed within the metaphase plate (Figure 2H, Figure 3G, Figure 4H and Figure 5G), or, conversely, chromosomes that appeared insufficiently condensed for this stage or already undergoing premature decondensation despite not having segregated to the poles (Figure 2I,J, Figure 3H,I, Figure 4I and Figure 5H).

In anaphase, chromatid separation appeared impaired, most notably in *V. faba*, where sticky telomeric ends were clearly visible at both early and late stages of anaphase (Figure 2K–N). Similar, though less distinct, abnormalities were also observed in the remaining species, likely due to their smaller chromosomes (Figure 3J, Figure 4J,K and Figure 5I–K). These segregation defects often resulted in the formation of chromosomal bridges or micronuclei during telophase (Figure 2O,P, Figure 3K,L, Figure 4K and Figure 5K), as well as in atypical patterns of chromatin condensation in telophase nuclei (Figure 4L and Figure 5L).

The percentage of mitotic figures exhibiting abnormalities or chromosomal aberrations was similar across all studied species, averaging around 20–25% of dividing cells (Figure 1A–D). The highest proportion of defects (slightly over 30%) was observed during metaphase (Figure 1(A′–D′)).

### 2.2. Assessment of the SEO Effects on Cell Cycle Regulator

**Figure 6:**
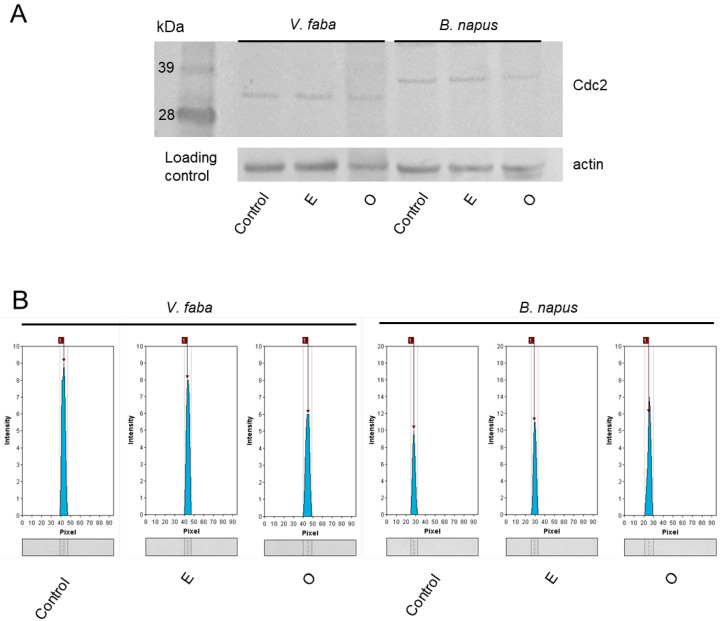
Western blot analysis of Cdc2 in protein extracts from the apical region of *V. faba* and *B. napus* roots after 24 h seedling incubation in water—Control, emulsifier solution—E, or emulsified SEO at the IC_50_ concentration—O (**A**). Loading control represents the level of actin detected between 49 and 38 kDa. Microdensitometric analysis of Cdc2 band intensity on the membrane, performed using the GelAnalyzer software, to compare relative protein abundance between treatments (**B**).

The observed decrease in mitotic activity prompted an analysis of the level of a key mitotic regulatory protein, Cdc2, whose presence is maintained throughout the cell cycle, in contrast to cyclins that undergo periodic synthesis and degradation. This choice allowed the detection of the protein regardless of the cell cycle phase. Western blot analysis focused on representatives of the Fabaceae and Brassicaceae families revealed that Cdc2 protein levels in root meristem cells of *V. faba* and *B. napus* were similar in the control and emulsifier-treated seedlings, with relative band intensities of 9 and 8 [a.u.] for *V. faba* and 10 and 11 [a.u.] for *B. napus*, respectively. Exposure to SEO at the IC_50_ concentration resulted in a marked decrease in Cdc2 abundance, with intensities reduced to 6 [a.u] in *V. faba* and 7 [a.u.] in *B. napus*, indicating that the essential oil inhibited the accumulation of this key cell cycle regulator (Figure 6).

### 2.3. Assessment of the SEO Effects on Plant Cytoskeleton

The observed disturbances in mitotic figures, particularly during metaphase and anaphase, suggested that defects in spindle organization might be responsible, as the dynamic reorganization of the mitotic spindle is essential for proper chromosome movement. Since the spindle is composed of polymerized heterodimers of α- and β-tubulin, which can be identified immunocytochemically, an analysis was undertaken to visualize β-tubulin using fluorescence microscopy.

**Figure 7:**
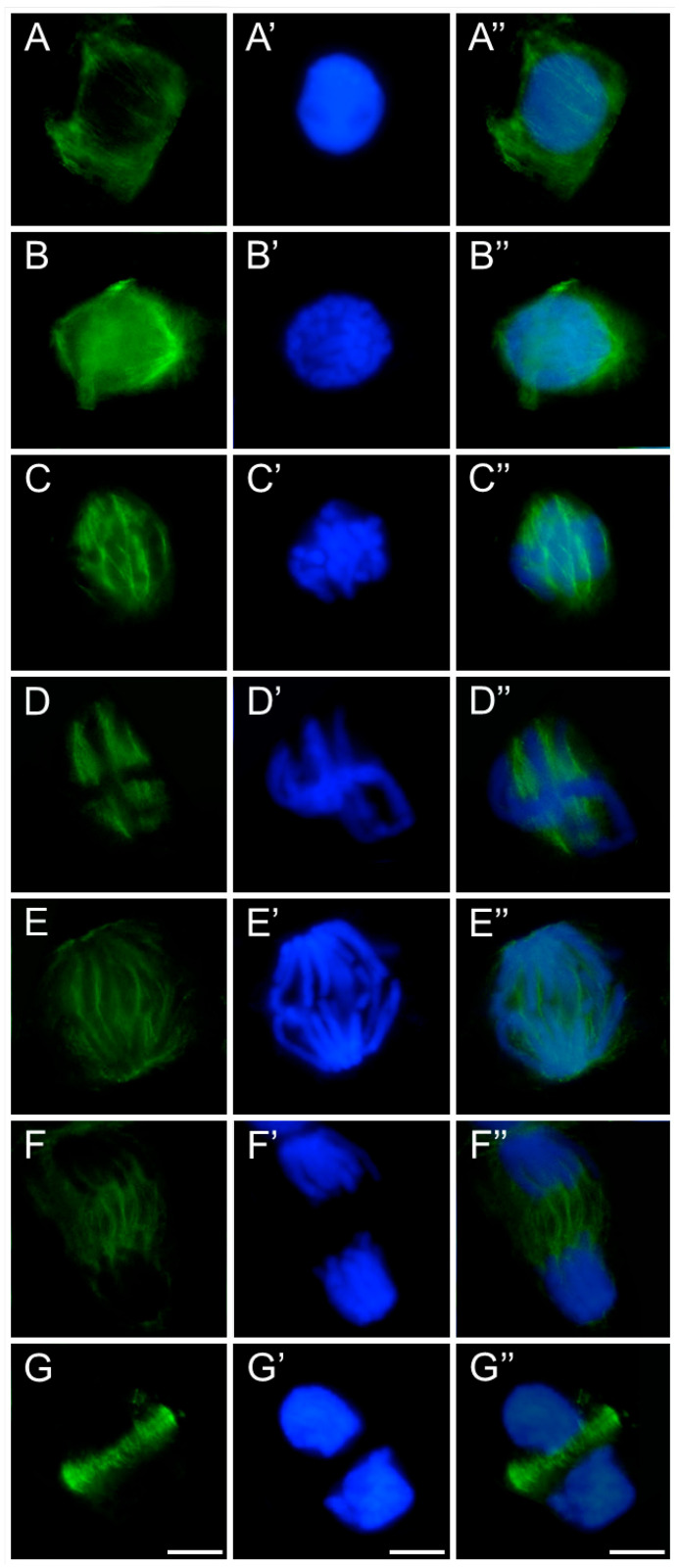
Immunocytochemical detection of β-tubulin (**A**–**G**) in root meristem cells of *V. faba* after 24 h seedling incubation in water—Control. Nuclei stained with DAPI (**A′**–**G′**), merged images (**A″**–**G″**). Scale bar: 10 µm.

**Figure 8:**
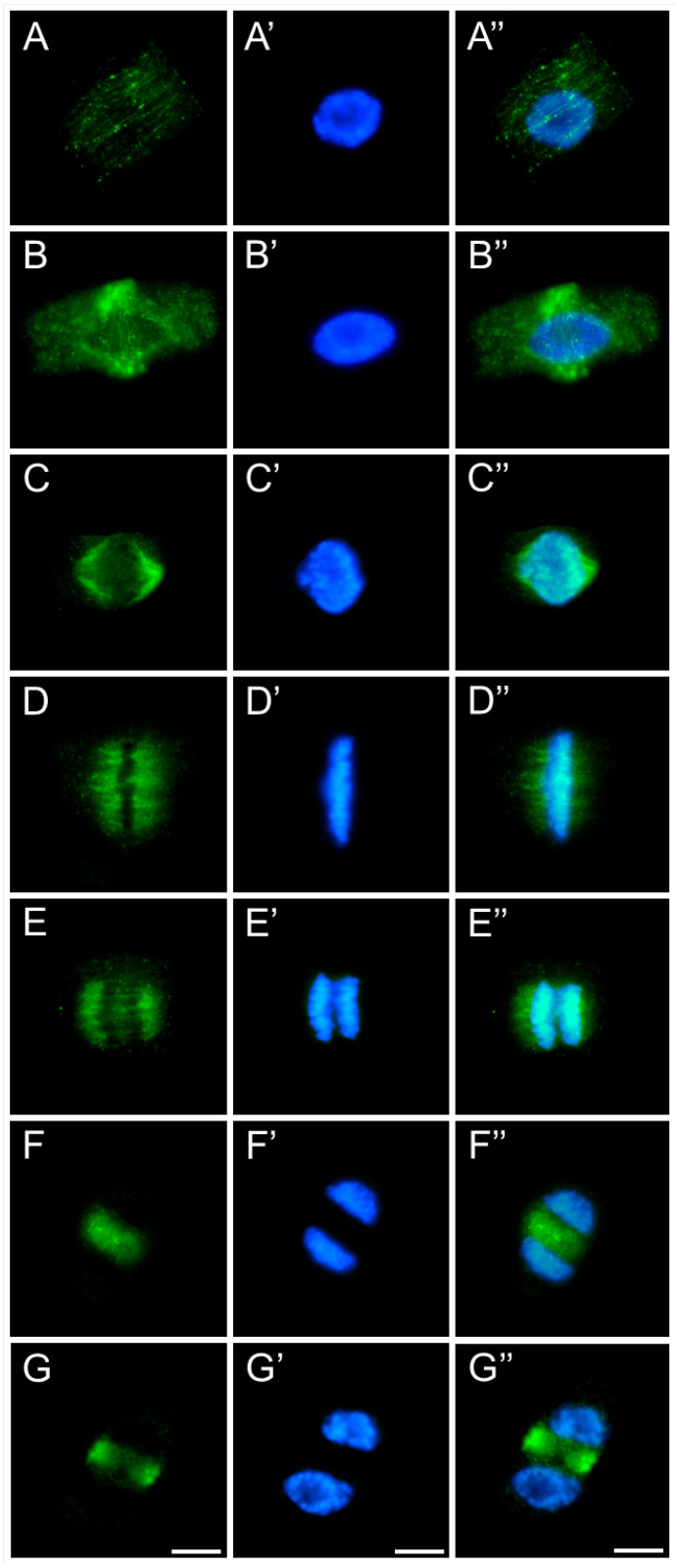
Immunocytochemical detection of β-tubulin (**A**–**G**) in root meristem cells of *L. luteus* after 24 h seedling incubation in water—Control. Nuclei stained with DAPI (**A′**–**G′**), merged images (**A″**–**G″**). Scale bar: 10 µm.

**Figure 9:**
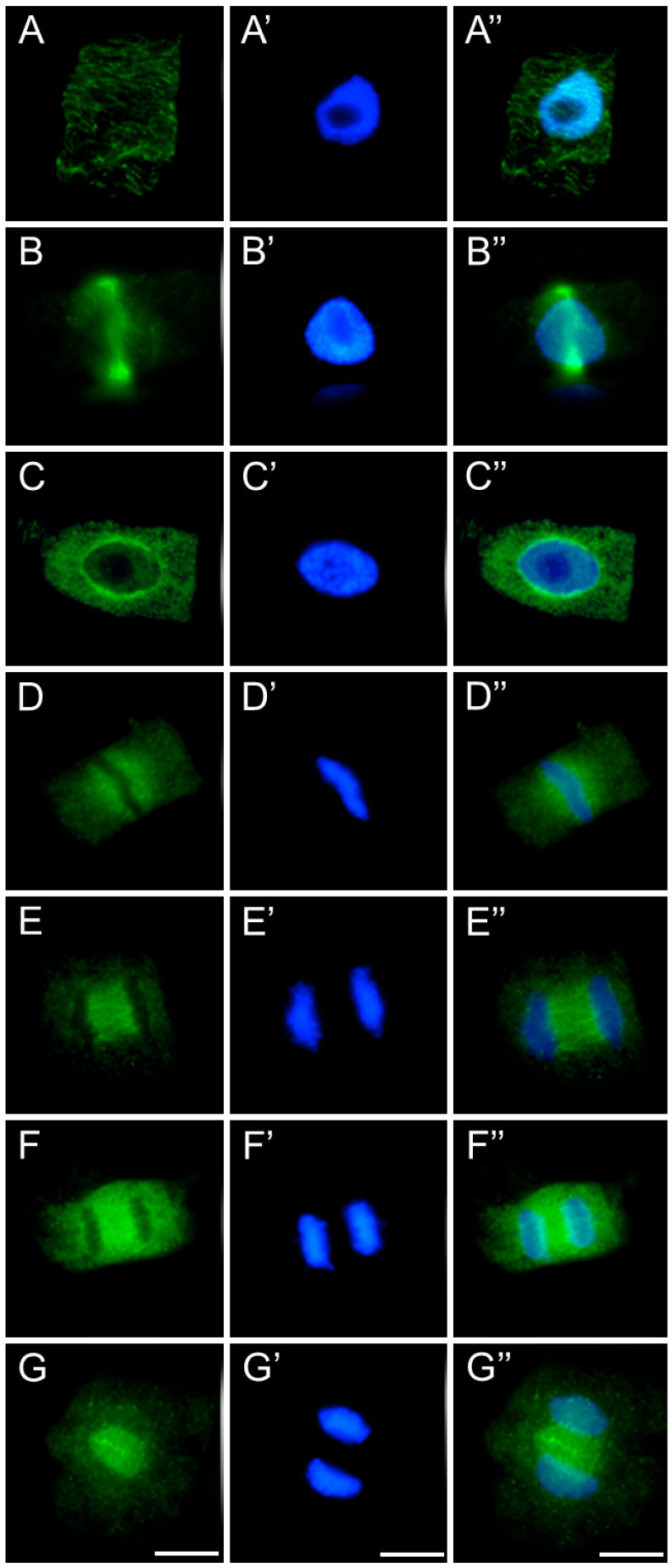
Immunocytochemical detection of β-tubulin (**A**–**G**) in root meristem cells of *B. napus* after 24 h seedling incubation in water—Control. Nuclei stained with DAPI (**A′**–**G′**), merged images (**A″**–**G″**). Scale bar: 10 µm.

**Figure 10:**
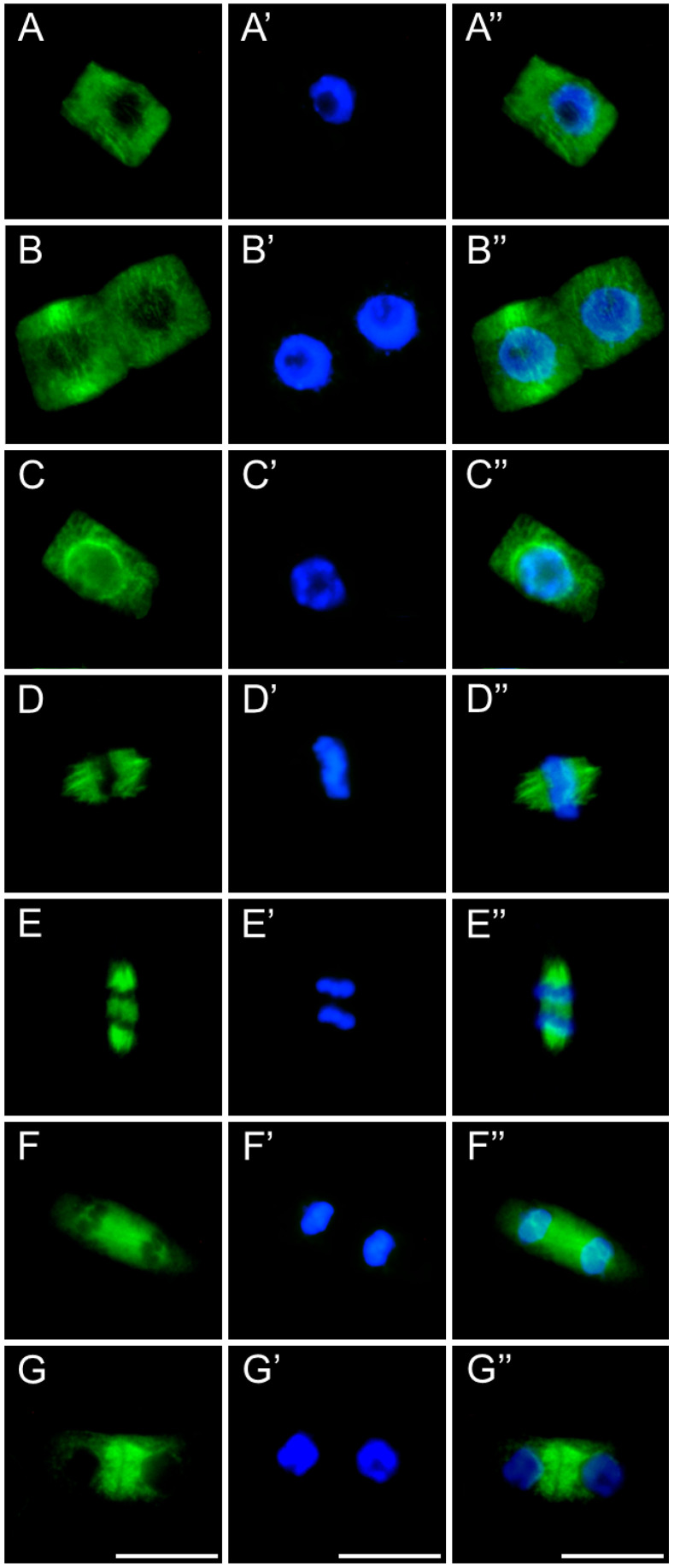
Immunocytochemical detection of β-tubulin (**A**–**G**) in root meristem cells of *A. thaliana* after 24 h seedling incubation in water—Control. Nuclei stained with DAPI (**A′**–**G′**), merged images (**A″**–**G″**). Scale bar: 10 µm.

In meristematic cells of control and emulsifier-treated roots of *V. faba* (Figure 7), *L. luteus* (Figure 8), *B. napus* (Figure 9), and *A. thaliana* (Figure 10), microtubules during interphase are organized into a cortical array composed of fine, delicate fibers (Figure 7, Figure 8, Figure 9 and Figure 10(A–A″)). In preprophase, they reorganize to form the preprophase band, a transient structure that marks the future division plane (Figure 7, Figure 8, Figure 9 and Figure 10(B–B″)). As this band disappears, microtubule material becomes concentrated at opposite regions of the nuclear envelope, where dispersed microtubule-organizing centers serve as nucleation sites for spindle assembly. In prophase, following nuclear envelope breakdown, chromosomes encounter microtubules nucleated from these dispersed centers (Figure 7, Figure 8, Figure 9 and Figure 10(B–B″,C–C″)). These microtubules, attached to kinetochores, form visible kinetochore fibers that align chromosomes at the metaphase plate and interlink to establish the spindle apparatus (Figure 7, Figure 8, Figure 9 and Figure 10(D–D″)). During anaphase, spindle microtubules depolymerize, enabling chromatid separation toward the poles, while sliding forces supported by polar microtubules in the central spindle contribute to pole separation (Figure 7, Figure 8, Figure 9 and Figure 10(E–E″)). Once chromatid segregation is completed in late anaphase (Figure 7, Figure 8, Figure 9 and Figure 10(F–F″)), remnants of the mitotic spindle give rise to the phragmoplast, a cytokinetic spindle (Figure 7, Figure 8, Figure 9 and Figure 10(G–G″)), which serves as a scaffold for cell plate formation. Seedlings treated with emulsifier showed no alterations in tubulin architecture compared to the control.

**Figure 11:**
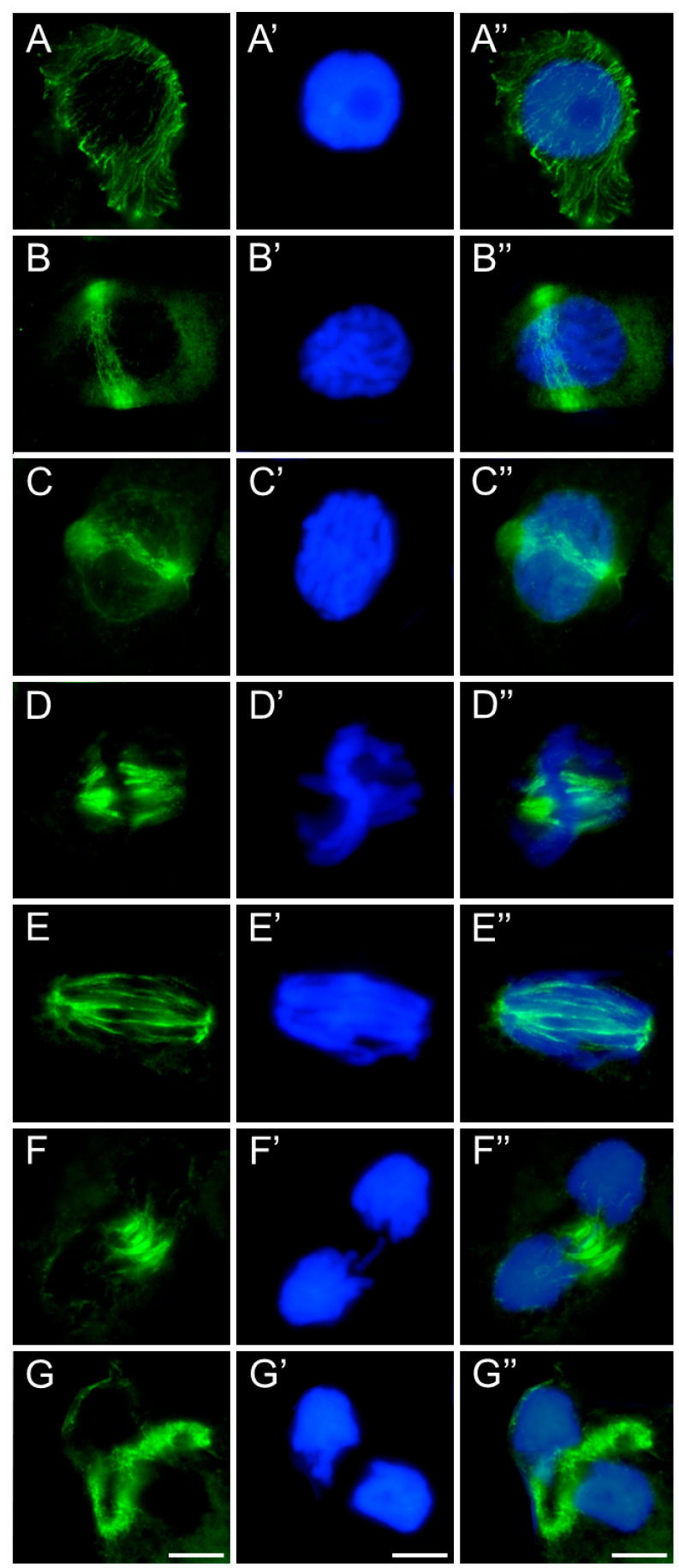
Immunocytochemical detection of β-tubulin (**A**–**G**) in root meristem cells of *V. faba* after 24 h seedling incubation in emulsified SEO at the IC_50_ concentration. Nuclei stained with DAPI (**A′**–**G′**), merged images (**A″**–**G″**). Scale bar: 10 µm.

**Figure 12:**
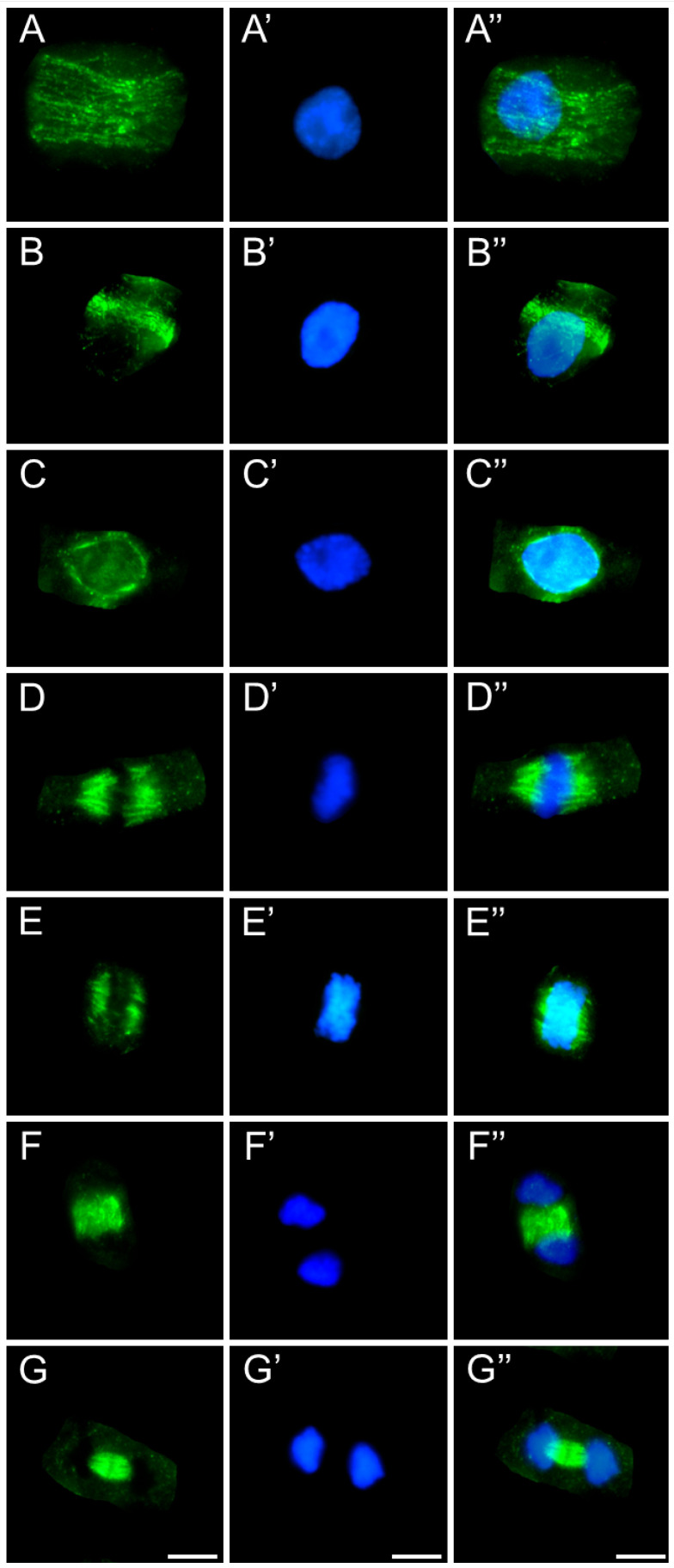
Immunocytochemical detection of β-tubulin (**A**–**G**) in root meristem cells of *L. luteus* after 24 h seedling incubation in emulsified SEO at the IC_50_ concentration. Nuclei stained with DAPI (**A′**–**G′**), merged images (**A″**–**G″**). Scale bar: 10 µm.

**Figure 13:**
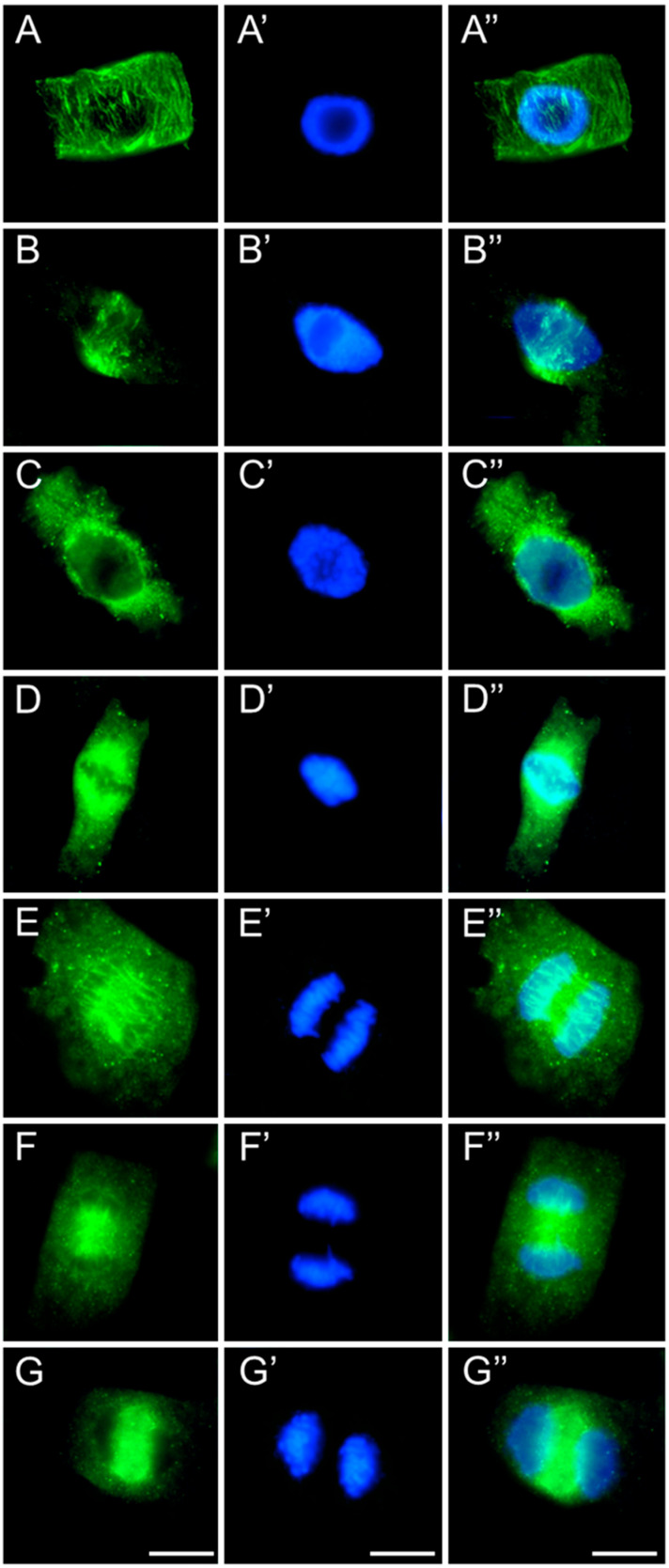
Immunocytochemical detection of β-tubulin (**A**–**G**) in root meristem cells of *B. napus* after 24 h seedling incubation in emulsified SEO at the IC_50_ concentration. Nuclei stained with DAPI (**A′**–**G′**), merged images (**A″**–**G″**). Scale bar: 10 µm.

**Figure 14:**
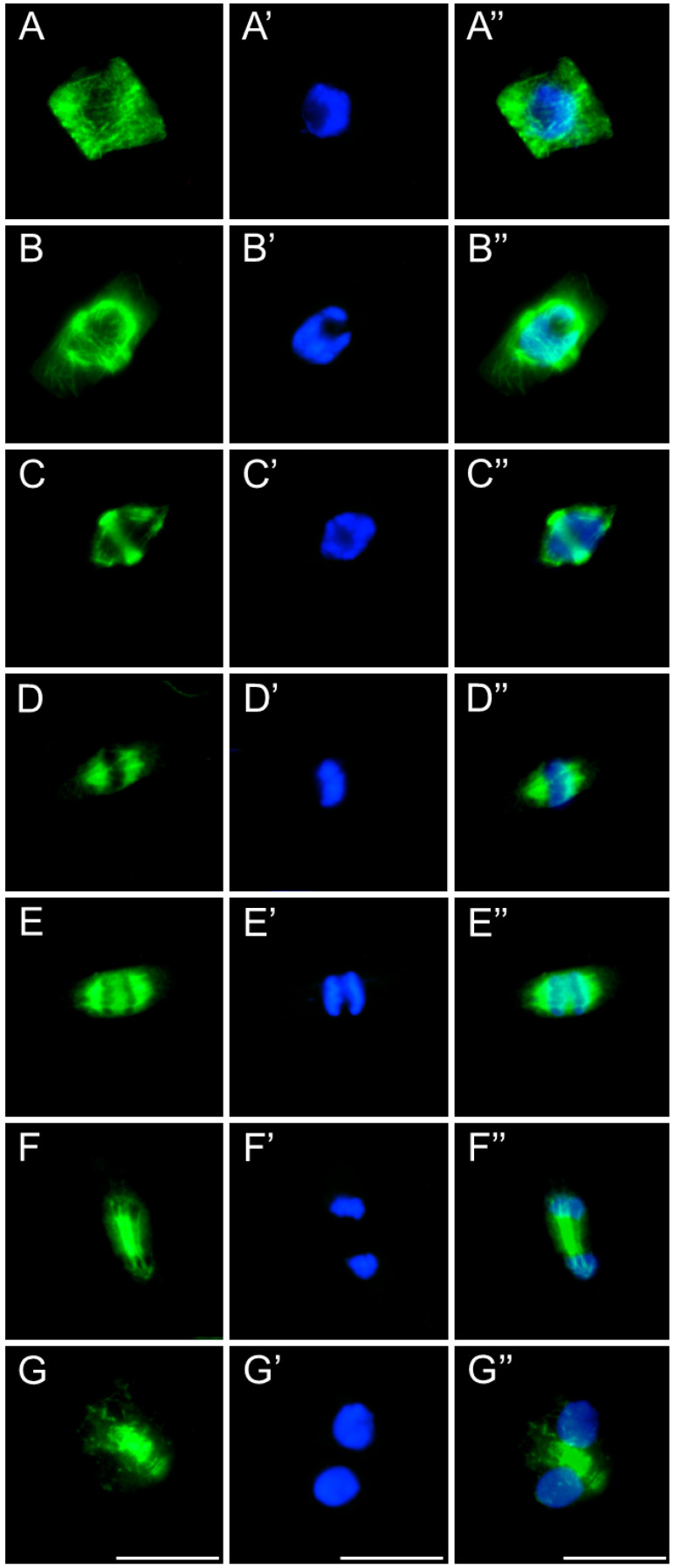
Immunocytochemical detection of β-tubulin (**A**–**G**) in root meristem cells of *A. thaliana* after 24 h seedling incubation in emulsified SEO at the IC_50_ concentration. Nuclei stained with DAPI (**A′**–**G′**), merged images (**A″**–**G″**). Scale bar: 10 µm.

After 24 h of SEO treatment, the tubulin architecture in *V. faba* (Figure 11), *L. luteus* (Figure 12), *B. napus* (Figure 13), and *A. thaliana* (Figure 14) was clearly disturbed, with comparable abnormalities observed in all tested species. In interphase cells, cortical microtubule fibers appeared noticeably thicker and more intensely fluorescent than the delicate, finely dispersed microtubule arrays seen in control cells, suggesting stronger lateral associations and enhanced stabilization (Figure 11, Figure 12, Figure 13 and Figure 14(A–A″)). In preprophase cells (Figure 11, Figure 12, Figure 13 and Figure 14(B–B″)), the preprophase band also showed a more robust, sometimes distorted structure, often persisting unusually long into late prophase without effectively providing building material for spindle formation at the poles (see also Figure 11, Figure 12, Figure 13 and Figure 14(C–C″)). In metaphase, the kinetochore fibers within the forming spindle appeared densely packed, consisting of tightly bundled microtubules anchored at the kinetochores, forming conspicuously thick strands (Figure 11, Figure 12, Figure 13 and Figure 14(D–D″)). During anaphase, especially in *V. faba*, polar microtubules were clearly visible, displaying the same massive, bundled organization, with distinct tubulin accumulations near the kinetochores already pulled toward the poles (Figure 11, Figure 12, Figure 13 and Figure 14(E–E″)). In telophase, the architecture of the phragmoplast was similarly affected, with prominent, robust microtubule bundles forming massive strands within the cytokinetic spindle (Figure 11, Figure 12, Figure 13 and Figure 14(F–F″,G–G″)).

**Figure 15:**
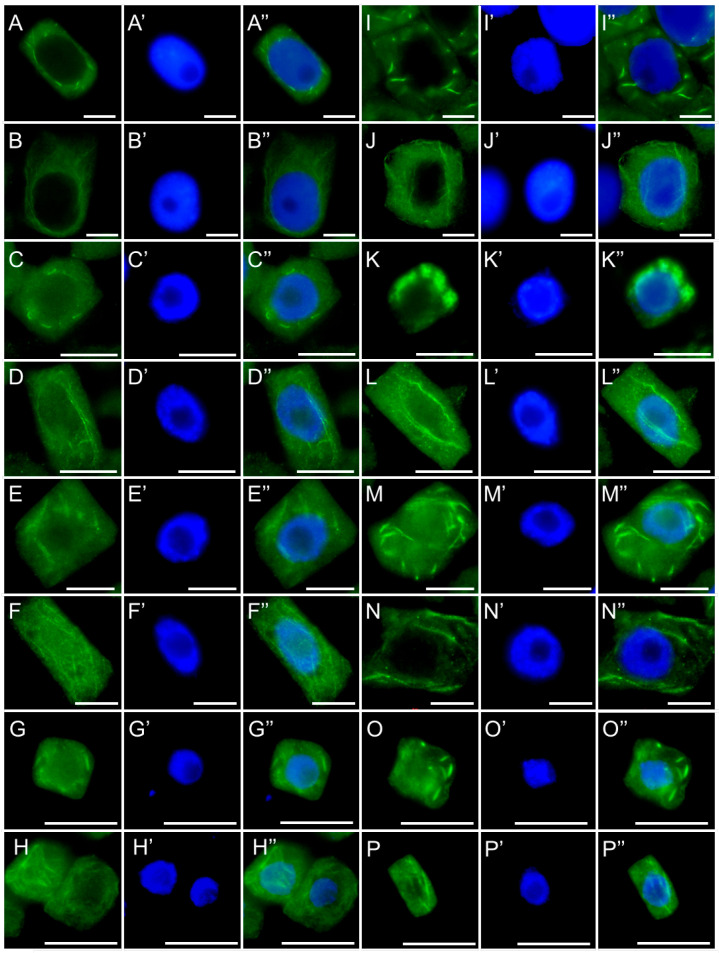
Immunocytochemical detection of actin (**A**–**P**) in root meristem cells of *V. faba* (**A**,**B**,**I**,**J**), *L. luteus* (**C**,**D**,**K**,**L**), *B. napus* (**E**,**F**,**M**,**N**), *A. thaliana* (**G**,**H**,**O**,**P**) after 24 h seedling incubation in water—Control (**A**–**H**) and in emulsified SEO at the IC_50_ concentration (**I**–**P**). *Panel descriptions are provided in the main text.* Nuclei stained with DAPI (**A′**–**P′**), merged images (**A″**–**P″**). Scale bar: 10 µm.

Another equally important component of the plant cell cytoskeleton is the actin network, which is highly responsive to various microlocal signals. In meristematic cells, the actin architecture undergoes dynamic reorganization, ranging from delicate, extended filaments forming a dispersed cortical network to more compact, locally concentrated bundles. In control cells and those treated with the emulsifier, both types of actin organization were observed in all four analyzed species (Figure 15(A–A″,C–C″,E–E″,G–G″)). After 24-hour exposure of *V. faba* (Figure 15(B–B″)), *L. luteus* (Figure 15(D–D″)), *B. napus* (Figure 15(F–F″)), and *A. thaliana* (Figure 15(H–H″)) seedlings to SEO at the IC_50_ concentration, a similar overall pattern was maintained; however, both the extended filaments and the compact bundles appeared thicker and more pronounced, exhibiting stronger fluorescence signals as a result.

### 2.4. Assessment of the Alterations in Chromatin Structure by Modifying H3 Phosphorylation Patterns Followed by SEO Treatment

The condensation of chromatin into mitotic chromosomes and the recruitment of specific mitotic regulatory proteins are fundamental events ensuring the accurate segregation of genetic material during mitosis. This process is tightly regulated by specific histone epigenetic modifications. Among the most common epigenetic marks associated with mitosis are the phosphorylation of histone H3 at threonine 3 and serine 10. In all examined species, similar defects in chromatin condensation and mitotic figure morphology were observed. Therefore, the epigenetic analyses focused on *V. faba* as a representative of the Fabaceae family and *B. napus* as a representative of the Brassicaceae.

**Figure 16:**
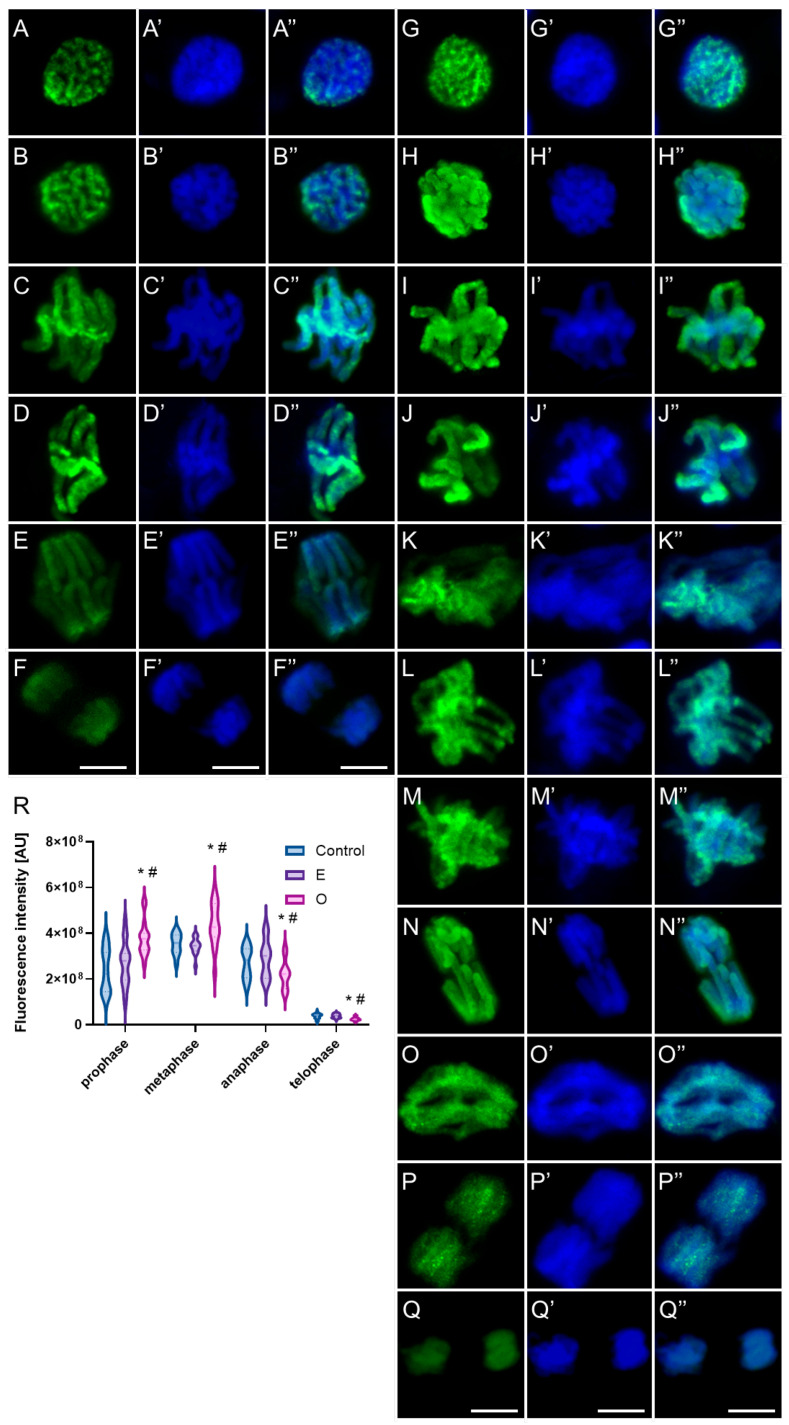
Immunofluorescence detection of H3T3Ph in root meristem cells of *V. faba* after 24 h seedling incubation in water—Control (**A**–**F**) and in emulsified SEO at the IC_50_ concentration (**G**–**Q**). Nuclei stained with DAPI (**A′**–**Q′**), merged images (**A″**–**Q″**). Panel descriptions are provided in the main text. Scale bar: 10 µm. Mean fluorescence intensity of the H3T3Ph signal in prophase, metaphase, anaphase and telophase root meristem cells of *V. faba* after 24 h seedling incubation in water—Control, the emulsifier solution—E, or emulsified SEO at the IC_50_ concentration—O, (**R**). The width of each violin plot represents the distribution of values along the y-axis; medians are indicated by dashed lines, and quartiles by dotted lines. Statistical differences were assessed using the Mann–Whitney test at *p* ≤ 0.05. Asterisk (*) and hash (#) marks denote significant differences compared to the control and the emulsifier, respectively.

**Figure 17:**
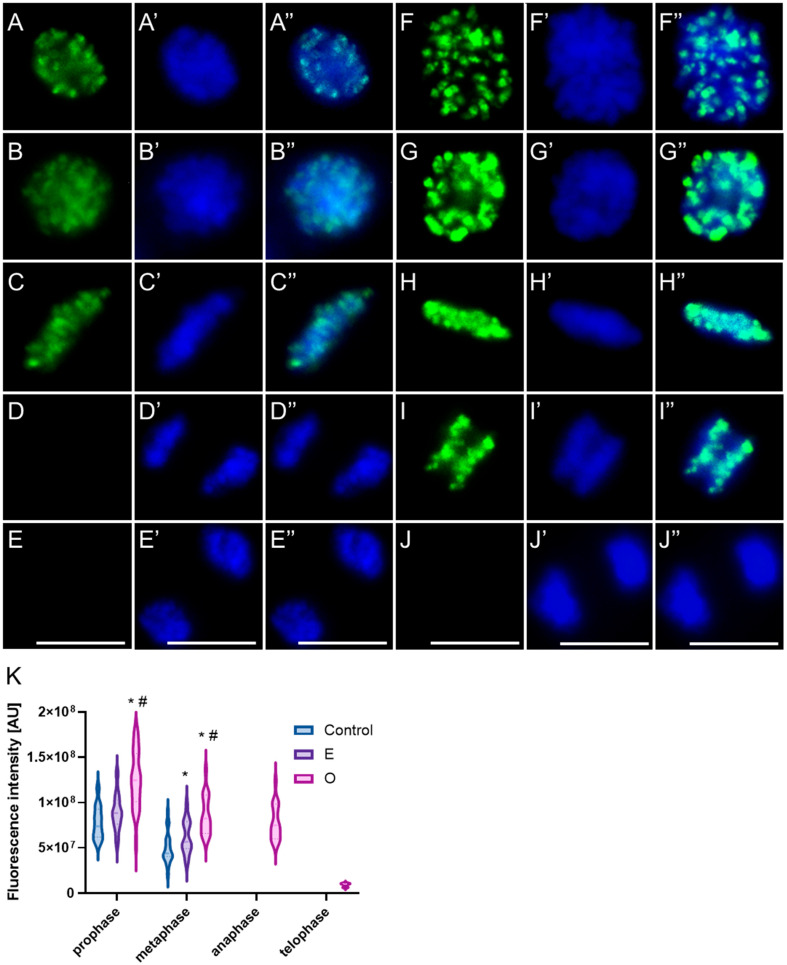
Immunofluorescence detection of H3T3Ph in root meristem cells of *B. napus* after 24 h seedling incubation in water—Control (**A**–**E**) and in emulsified SEO at the IC_50_ concentration (**F**–**J**). Nuclei stained with DAPI (**A′**–**J′**), merged images (**A″**–**J″**). Panel descriptions are provided in the main text. Scale bar: 10 µm. Mean fluorescence intensity of the H3T3Ph signal in prophase, metaphase, anaphase and telophase root meristem cells of *B. napus* after 24 h seedling incubation in water—Control, the emulsifier solution—E, or emulsified SEO at the IC_50_ concentration—O, (**K**). The width of each violin plot represents the distribution of values along the y-axis; medians are indicated by dashed lines, and quartiles by dotted lines. Statistical differences were assessed using the Mann–Whitney test at *p* ≤ 0.05. Asterisk (*) and hash (#) marks denote significant differences compared to the control and the emulsifier, respectively.

In mitotic root cells of control plants (*V. faba* and *B. napus*), the phosphorylated form of histone H3 at threonine 3 (H3T3Ph) appeared on early prophase chromosomes as discrete immunopositive foci (Figure 16 and Figure 17(A–A″)). The number and intensity of these foci progressively increased as chromatin condensation advanced, culminating in a strong pericentromeric signal during late prophase, accompanied by weaker labeling along the chromosome arms (Figure 16 and Figure 17(B–B″)). Upon chromosome alignment at the metaphase plate, the intensely labeled centromeric regions were positioned at the central plane, while the distinctly condensed metaphase chromosomes displayed a mosaic labeling pattern along the arms, with several foci of higher intensity, including enhanced fluorescence at the telomeric regions. However, due to interspecies differences in chromosome number and morphology, particularly in chromosome length, the overall labeling pattern in prophase and metaphase appeared slightly different between *V. faba* and *B. napus* (Figure 16(C–C″,D–D″) and Figure 17(C–C″)). Once sister chromatids began to separate during anaphase, the H3T3Ph signal markedly diminished in *V. faba* (Figure 16(E–E″)) and disappeared entirely in *B. napus* (Figure 17(D–D″)), leaving telophase chromosomes devoid of specific detectable labeling (Figure 16(F–F″) and Figure 17(E–E″)).

Following treatment with SEO at the IC_50_ concentration, H3T3Ph-associated fluorescence signals were generally markedly enhanced in mitotic cells of both *V. faba* and *B. napus*, with increased signal intensity and expanded distribution of immunopositive foci (Figure 16(G–P,G′–P′,G″–P″,R) and Figure 17(F–I,F′–I′,F″–I″,K)). However, the mitotic cell population displayed heterogeneous chromatin condensation patterns in particular phases, which corresponded to variable fluorescence labeling profiles (especially evident in *V. faba* due to large chromosomes, as shown in the panel; similar effects were seen in *B. napus*, but not imaged). In metaphase cells with strongly condensed chromosomes, intense fluorescence was observed along the chromosome arms, often with enhanced signals at telomeric regions (Figure 16(J–J″). In contrast, cells exhibiting atypically low condensation of metaphase chromosomes showed a more diffuse and extended labeling pattern, lacking the characteristic pericentromeric enrichment seen in control cells, and displaying a markedly reduced mosaic signal along the arms and telomeres (Figure 16(K–K″,L–L″)). Moreover, in both species, while structurally normal anaphases were observed, a subset of cells exhibiting structural abnormalities during anaphase showed persistent strong H3T3Ph labeling into early anaphase (Figure 16(M–M″)). In later stages of anaphase, this signal was weaker and primarily restricted to the pericentromeric regions or remained visible along the arms of the chromosomes (Figure 16(N–N″,O–O″)). During telophase, residual H3T3Ph labeling was occasionally detectable as small discrete foci distributed along the chromosome arms in a fraction of cells (compare Figure 16(P–P″,Q–Q″)).

**Figure 18:**
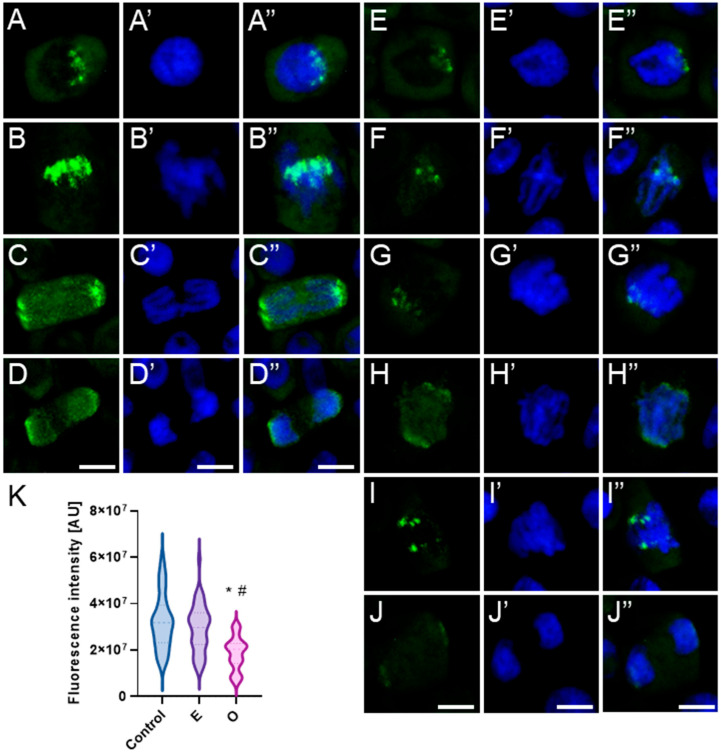
Immunofluorescence detection of H3S10Ph in root meristem cells of *V. faba* after 24 h seedling incubation in water—Control (**A**–**D**) and in emulsified SEO at the IC_50_ concentration (**E**–**J**). Nuclei stained with DAPI (**A′**–**J′**), merged images (**A″**–**J″**). Panel descriptions are provided in the main text. Scale bar: 10 µm. Mean fluorescence intensity of the H3S10Ph signal in prophase, metaphase, anaphase and telophase root meristem cells of *V. faba* after 24 h seedling incubation in water—Control, the emulsifier solution—E, or emulsified SEO at the IC_50_ concentration—O (**K**). The width of each violin plot represents the distribution of values along the y-axis; medians are indicated by dashed lines, and quartiles by dotted lines. Statistical differences were assessed using the Mann–Whitney test at *p* ≤ 0.05. Asterisk (*) and hash (#) marks denote significant differences compared to the control and the emulsifier, respectively.

**Figure 19:**
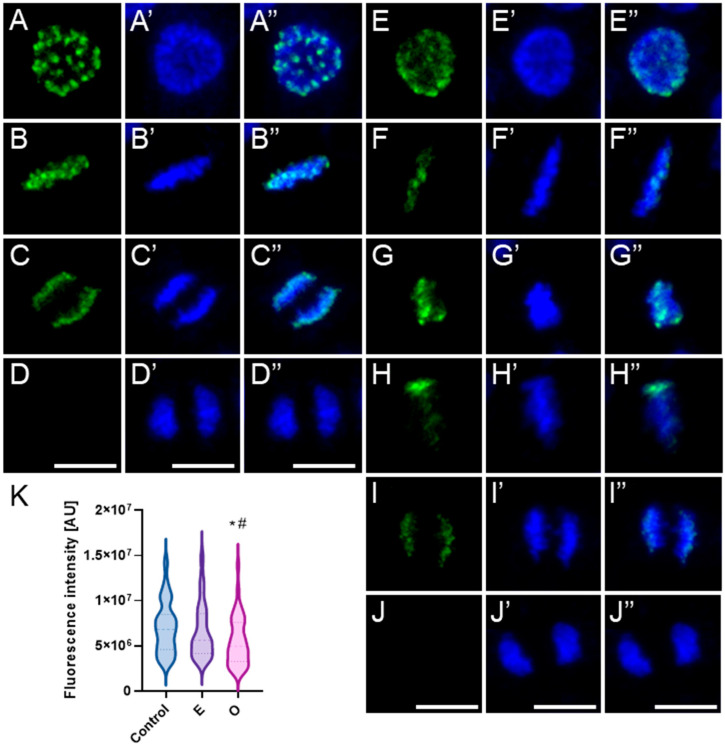
Immunofluorescence detection of H3S10Ph in root meristem cells of *B. napus* after 24 h seedling incubation in water—Control (**A**–**D**) and in emulsified SEO at the IC_50_ concentration (**E**–**J**). Nuclei stained with DAPI (**A′**–**J′**), merged images (**A″**–**J″**). Panel descriptions are provided in the main text. Scale bar: 10 µm. Mean fluorescence intensity of the H3S10Ph signal in prophase, metaphase, anaphase and telophase root meristem cells of *B. napus* after 24 h seedling incubation in water—Control, the emulsifier solution—E, or emulsified SEO at the IC_50_ concentration—O (**K**). The width of each violin plot represents the distribution of values along the y-axis; medians are indicated by dashed lines, and quartiles by dotted lines. Statistical differences were assessed using the Mann–Whitney test at *p* ≤ 0.05. Asterisk (*) and hash (#) marks denote significant differences compared to the control and the emulsifier, respectively.

Under physiological conditions, the phosphorylated form of histone H3 at serine 10 (H3S10Ph) is predominantly localized to the pericentromeric regions of mitotic chromosomes (Figure 18 and Figure 19(A–D,A′–D′,A″–D″)). The signal first appears in early prophase, typically as intensely fluorescent foci clustered at one pole of the nucleus. In species with a high DNA content, such as *Vicia faba*, this distribution reflects the classical Rabl configuration, in which chromosomes maintain a polarized orientation inherited from the previous mitotic division (Figure 18(A–A″)). In contrast, in species with lower DNA content, such as *Brassica napus*, the H3S10Ph signal may be more evenly distributed across the entire prophase nucleus, without clear polarization (Figure 19(A–A″)). In metaphase, the fluorescent signal aligns along the metaphase plate, corresponding to the position of centromeres arranged in the division plane (Figure 18 and Figure 19(B–B″)). During anaphase, the labeled centromeric regions migrate toward opposite poles, consistent with the movement of centromeres being pulled first by the spindle microtubules (Figure 18 and Figure 19(C–C″)). In telophase, as chromatin begins to decondense, the intensity of the H3S10Ph signal gradually diminishes—remaining faintly detectable in some cells, but typically disappearing entirely in later stages (Figure 18 and Figure 19(D–D″)). This characteristic labeling pattern was observed in both control cells and cells treated with the emulsifier in root meristems of *V. faba* and *B. napus*.

Following treatment with SEO at the IC_50_ concentration, no changes in the localization of the H3S10Ph signal were observed in root meristematic cells of either species (Figure 18 and Figure 19(E–J,E′–J′,E″–J″)). Fluorescent foci appeared in the same chromosomal regions as under control conditions; however, the overall signal intensity was markedly reduced in *V. faba* and slightly less so in *B. napus* (Figure 18K and Figure 19K). Moreover, metaphase and early anaphase chromosomes exhibiting exceptionally strong condensation emitted a more intense fluorescence signal (Figure 18(I–I″) and Figure 19(G–G″)), whereas in metaphase chromosomes with atypically weak condensation, the H3S10Ph signal was faint or even undetectable (Figure 18(G–G″) and Figure 19(H–H″)).

## 3. Discussion

Plant growth, from the earliest stages of development through to the end of the life cycle, is sustained by the continuous activity of meristematic tissues. Consequently, cell cycle is at the core of current research efforts aimed at identifying effective strategies to suppress the growth of undesirable plants, thereby enhancing the protection and productivity of crop species in monoculture systems [41]. In response to the growing demand for environmentally safe plant protection agents, increasing attention has been directed toward natural product-based alternatives [25,28,32,95,96]. In our study, which focused on evaluating the bioherbicidal potential of essential oil extracted from the rhizomes of sweet flag (*A. calamus*, SEO), we also investigated its impact on the progression of the plant cell cycle.

### 3.1. Bioherbicidal Potential of SEO: Root Growth Inhibition via Cell Cycle Disruption

**Figure 20:**
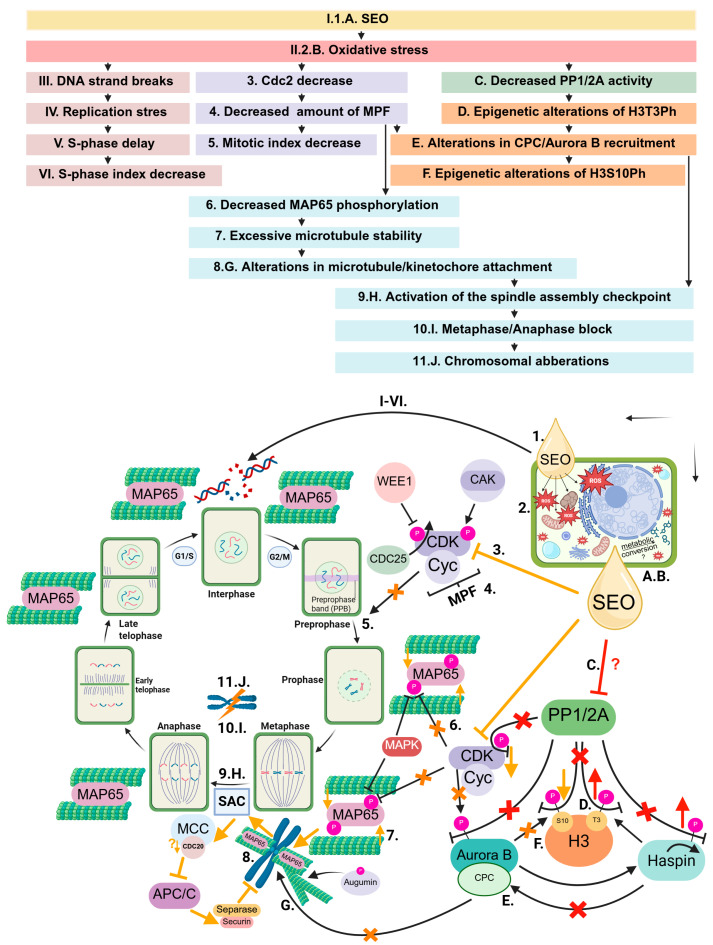
Effect of SEO on meristematic cell proliferation: ROS generation and DNA break formation lead to a slowdown of S-phase progression (pathways I–VI), mitotic entry arrest, and mitotic defects through decreased levels of cyclin-dependent kinase Cdc2 (the conserved plant CDKA homolog) and alterations in microtubule dynamics (pathways 1–11), as well as H3 histone epigenetic disturbances, likely associated with reduced PP1/2A phosphatase activity (pathways A–J). The pathway numbering used in the flowchart is maintained in the illustrative panel depicting the processes discussed. A detailed description of the events is provided in the Introduction and Discussion, and abbreviations are explained in the “Abbreviations” section. Orange arrows indicate changes resulting from cyclin-dependent kinase deficiency, with orange X marks representing the processes affected. Red arrows and X marks similarly denote effects of PP1/2A activity inhibition. Created with biorender.com.

Our previous research demonstrated that SEO exerts a species-specific, concentration-dependent phytotoxic effect, limiting Brassicaceae growth while sparing Fabaceae. This effect was associated with oxidative stress: Fabaceae accumulated higher ROS levels but activated more efficient antioxidant defenses, whereas Brassicaceae, despite lower ROS accumulation, experienced stronger metabolic disruptions [22]. In root meristems, this oxidative stress triggered replication stress, causing DNA double-strand breaks. Consequently, the replication index dropped to 70% of control levels in Fabaceae and 80% in Brassicaceae, and replication dynamics decreased to 80% and 70% of control, respectively. While root meristem cells exhibited a unified pattern of response to SEO-induced stress, their sensitivity varied across species. These early effects on the cell cycle were followed, as confirmed in the present study, by disruptions in mitotic progression (Figure 20). SEO significantly reduced cell proliferation, as reflected by a 50–60% decrease in the mitotic index, promoted the accumulation of cells at metaphase, and induced mitotic aberrations, including irregular chromatin organization and structural defects in karyokinetic and cytokinetic spindles. The reduction in mitotic index may partly result from the accumulation of cells in G1, the reduced proportion of cells entering S phase, and impaired replication dynamics associated with DNA damage, including difficulties in replicating highly condensed heterochromatin, as indicated by an increased proportion of cell in the late S phase [41]. However, the pronounced inhibition of mitotic activity and the structural abnormalities observed in dividing cells indicate multifaceted interference with key, interdependent mechanisms regulating mitosis. Notably, similar effects, specifically a decrease in mitotic activity, were reported by Rajamanikkam et al. [42] in *Allium cepa* root tips treated with an aqueous extract of *A. calamus*, although without observable chromosomal abnormalities. Given the complex composition of SEO, as characterized by others [81] and in our previous study [91], including major and minor constituents that may act synergistically, it remains challenging to attribute these effects to specific compounds. Nevertheless, the high content of phenylpropanoids, particularly asarones, known for their strong biological activity, is likely to contribute substantially to the observed phytotoxic and cell cycle–modulating effects [40]. 

### 3.2. Underlying Mechanisms of Mitotic Index Reduction

Our previous studies revealed that SEO, aside from asarones, consists of a number of phenylpropanoid compounds, including methyl-eugenol, safrol, isoacoramone, and asaronaldehyde [97]. However, comprehensive studies examining the effects of these compounds across both plant and animal models remain limited, and the existing data do not yet provide a complete picture of their biological activity. In the present study, Western blot analysis revealed that SEO treatment led to a noticeable decrease in the abundance of the cell cycle regulator CDKA, detected using anti-Cdc2 antibodies (the conserved homolog of plant CDKA) in root apices of both *V. faba* and *B. napus*. Given that Cdc2 protein levels are generally maintained at a relatively constant level throughout the cell cycle, unlike cyclins whose abundance fluctuates, the observed reduction suggests either enhanced proteolytic degradation or decreased synthesis of this key mitotic kinase.

It is well established that phenylpropanoid compounds strongly affect plant cell cycle regulation. For instance, in *A. thaliana*, eugenol was shown to inhibit growth by upregulating stress-response genes while downregulating those required for mitotic progression, including CYCB which promotes entry into mitosis, TPX2 involved in microtubule organization and spindle assembly, and CDC20.1 required for sister chromatid separation [98,99]. Eugenol also suppressed genes involved in the biosynthesis of cell wall components (lignin, cellulose, hemicellulose, and pectin), essential for proper cell plate formation during cytokinesis [99]. Phenyl carboxylic derivatives of phenylpropanoids, such as benzoic and cinnamic acid derivatives, similarly inhibited root growth in germinating cucumber seeds by downregulating key cell cycle genes, including CDKA, CDKB, and several cyclins (CycA, B, D3;1, D3;2) [100]. Additional evidence for the antiproliferative potential of phenylpropanoids comes from studies on cinnamaldehyde and its analogs, which induced G2 arrest in human cancer cells by downregulating key G2/M regulators (CDK1, CDC25C, MAD2, CDC20). This was accompanied by CycB1 accumulation, which in the absence of active CDK1 failed to trigger mitotic entry [101]. Although the direct impact of asarones on gene expression and regulatory mechanisms in dividing plant meristematic cells remains unclear, their antiproliferative activity is well established in cancer models [35]. For example, Li et al. [102] demonstrated that β-asarone induces G1 arrest in glioma cells by upregulating the CKIs (p21 and p27), while downregulating key activators such as CDC25A, CDK2, Cyc D, and E.

These findings provide a rationale to suggest that, given the universal nature of the core regulatory mechanisms governing the cell cycle across plant, animal, and even cancer cells, the components of SEO may contribute to broad changes in gene expression in meristematic cells, beyond the observed reduction in Cdc2 (CDKA) abundance (Figure 20). The potential repression of cell cycle activators or the upregulation of inhibitors aligns with our observations—namely, the reduction in the proportion of mitotic cells, which could result from the failure to assemble an active mitosis-promoting factor (MPF), whose formation depends on the precise interaction of specific cyclins and kinases. At the same time, it is important to consider that SEO may interfere with the multistep activation of pre-existing CDK/cyclin complexes, a process that relies on the coordinated activities of WEE1 kinase, CAK (itself a cyclin—CDK complex), and CDC25-like phosphatases, which are further regulated by PP1 and PP2A phosphatases (Figure 20) [103]; SEO may potentially inhibit PP1/2A activity, as further discussed in relation to histone H3 epigenetic modifications. We are only at the early stage of uncovering the mechanism of SEO action, yet it is evident that it targets the intricate, multilayered regulatory network controlling cell proliferation, where disruption of even a single component may propagate through entire cascades of signaling events.

### 3.3. Disruption of Mitotic Progression via Cytoskeletal Disorganization Induced by SEO

A second clear target of SEO activity was the disruption of cells that had already passed the G2 checkpoint and entered division. The marked increase in the metaphase index indicates interference with spindle microtubule function, similar to the effects of classical microtubule-disrupting agents such as colchicine, oryzalin, and propyzamide [104].

This hypothesis was further supported by immunofluorescence analysis with anti-tubulin antibodies, which revealed major alterations in microtubule organization. Microtubules appeared thickened, densely bundled, and formed distinct bands, suggesting excessive lateral association and stabilization within the spindle structure. Additionally, difficulties in microtubule reorganization were observed, including the prolonged persistence of the preprophase band, even though nuclear morphology indicated that cells had already entered prometaphase—a stage that, under control conditions, typically coincides with the rearrangement of the microtubule network around the disassembling nuclear envelope [83].

Similarly, immunolabeling of actin microfilaments revealed enhanced fluorescence, indicating thickening, excessive stabilization, or aggregation that could impair proper reorganization during mitosis under SEO treatment. Despite these alterations, the general architecture of the actin cytoskeleton remained preserved. Short, bundled microfilaments and long strands stabilizing the nucleus in interphase were still present, along with the preprophase ring, the cage-like structure around the metaphase spindle, and short filaments in the phragmoplast. One potential target of SEO action may be the plant actin-binding protein AtFormin14 (AtFH14), which in *A. thaliana* links microfilaments and microtubules, facilitating proper spindle organization [105]. It is noteworthy that disturbances in actin organization in response to phenylpropanoids have not yet been reported in plant cells, making definitive conclusions difficult. Some insights come from the study of Sarheed et al. [85], in which the effects of various mint essential oils on the cytoskeleton of *A. thaliana* were evaluated. The authors reported that *Agastache rugosa* oil caused microtubule degradation, while *Mentha longifolia* had no effect on microtubules but led to actin elimination. In earlier work [20], neither *A. rugosa* oil nor its constituents (menthone and isomenthone) eliminated actin filaments, but rather induced their bundling into thick cables. Since these compounds are monoterpenes, this highlights that cytoskeletal responses may vary considerably even within the same chemical class.

However, disruptions in microtubule organization involving tubulin aggregation were also observed by Hermes et al. [106] in human hepatocellular carcinoma cells (HepG2) exposed to asarones and their epoxide metabolites. Although the overall microtubule network remained intact, its spatial organization was clearly altered, leading to tubulin condensation. Similar findings were reported by Nagle et al. [101], who demonstrated tubulin aggregation in colorectal cancer cells (HCT 116) treated with cinnamaldehyde, another phenylpropanoid compound. As suggested by Guo et al. [99], such cytoskeletal abnormalities may result from the downregulation of genes encoding spindle-associated proteins, such as TPX2. However, the direct effect of SEO constituents on the activity of cytoskeleton-associated proteins involved in microtubule remodeling cannot be ruled out.

Since the mitotic spindle still formed, it is unlikely that SEO interfered with proteins responsible for microtubule nucleation, such as the γ-tubulin ring complex (γ-TuRC), kinesin-14 motor proteins transporting γ-TuRC to the spindle poles, or α-Aurora kinase, which phosphorylates γ-TuRC in a complex with TPXL3 to promote its spindle association [107]. Similarly, RUNKEL kinase, which contains a microtubule-binding domain and participates in phragmoplast expansion during cytokinesis, appears to remain active [108].

A likely target of SEO is the MAP65 family of microtubule-associated proteins, which mediate lateral bundling of microtubules into parallel arrays and stabilize the mitotic spindle (Figure 20) [109]. In *A. thaliana*, the MAP65-1 binds to microtubules during interphase, anaphase, and telophase, but not during prophase or metaphase. Its function is tightly regulated by CDK and MAPK kinases [83,109]. Phosphorylation of MAP65-1 inhibits its microtubule-binding, while lack of phosphorylation leads to excessive accumulation and stabilization of microtubules, particularly in the central region of the metaphase spindle, delaying anaphase onset [109]. It is therefore highly probable that SEO, by affecting the expression of genes encoding cyclins and CDK kinases or by interfering with their activation, could have impaired or significantly reduced MAP65 phosphorylation, resulting in its sustained activity throughout mitosis. Consequently, prolonged lateral bundling of microtubules and impaired spindle remodeling may have occurred (Figure 20). The formation of excessively dense microtubule bundles could also involve disruptions in augmin function, which mediates branched microtubule nucleation and dense network formation [86]. Although direct evidence in plants is lacking, by analogy with animal cells, augmin’s interaction with microtubules may be regulated by phosphorylation via CDK or α-Aurora kinases [110]. These hypotheses will be tested experimentally in the next phase of our research.

Improperly formed, laterally bundled microtubule arrays may have hindered correct kinetochore attachment, potentially activating the spindle assembly checkpoint (SAC) at the metaphase/anaphase transition and sustaining activity of the mitotic checkpoint complex (MCC), which binds and inhibits CDC20, thereby preventing the activation of the anaphase-promoting complex/cyclosome (APC/C) [83]. Moreover, as demonstrated for eugenol [99], SEO components may also reduce the expression of genes encoding CDC20, further reinforcing APC/C inhibition. As a result, cells remain arrested in metaphase until the defect is resolved. This mechanism may explain the observed increase in the metaphase index. This interpretation is also supported by microscopic observations of partially decondensed chromatids remaining closely associated during metaphase, suggesting that cohesin was likely not degraded due to separase inhibition by securing in the absence of active APC/C (Figure 20) [88].

### 3.4. Disruption of Mitotic Progression via Epigenetic Modifications Induced by SEO

In a subpopulation of mitotic cells, the elevated metaphase index was accompanied by characteristic chromosomal aberrations. Highly condensed prophase foci localized to telomeric regions of sister chromatids persisted into metaphase and acted as ‘sticky ends’ in anaphase, preventing full separation during telophase. This resulted in chromatin bridges that subsequently formed micronuclei. Such stickiness may arise from volatile compounds denaturing nuclear proteins and impairing segregation, or from disrupted chromatin folding and organization [28].

Alternatively, the SEO-induced increase in the metaphase index, and associated mitotic abnormalities may stem from impaired activity of PP1 and PP2A phosphatases (Figure 20). These enzymes act throughout the cell cycle, coordinating chromatin condensation, mitotic kinase activity, microtubule organization, and the metaphase–anaphase transition. While their suppression is required to initiate mitosis, timely reactivation is essential for successful progression through later stages, particularly metaphase and anaphase [111,112]. The hypothesis that SEO inhibits PP1/2A is supported by results obtained with okadaic acid, a specific inhibitor of these phosphatases, which induces comparable mitotic defects in *V. faba* [112]. Further support comes from SEO-induced disruptions in conserved histone H3 phosphorylation marks (H3T3Ph, H3S10Ph) affecting both their levels and distribution.

Phosphorylation of H3T3 by Haspin kinase is essential for proper chromosome condensation and spindle assembly checkpoint function, as it recruits the chromosomal passenger complex (CPC) containing Aurora B. The CPC corrects kinetochore–microtubule attachment errors, ensures accurate chromosome segregation, and later contributes to spindle disassembly [83,84,88,113]. Aurora B also catalyzes phosphorylation of H3S10. While the regulation of Haspin in plants remains unclear, studies in other organisms suggest control by Polo-like kinases (Plk1, absent in plants), a feedback interaction with the CPC/Aurora B complex, and Haspin autophosphorylation [114,115], with inactivation mediated by PP1 [116]. Aurora B is likely activated by mitotic CDKs and inactivated by PP1 and PP2A, which stabilize kinetochore–microtubule attachments [117]. Thus, precise temporal and spatial regulation of both kinases is critical for correct chromatin dynamics during mitosis (Figure 20) [118].

In this study, SEO markedly altered the phosphorylation patterns of H3T3 and H3S10, indicating disruption of epigenetic regulation of mitosis. H3T3Ph showed stronger and more widely distributed signals, persisting abnormally into anaphase and telophase. This likely reflects impaired PP1/PP2A activity, leading to sustained Haspin activation and insufficient H3T3 dephosphorylation [116]. In contrast, H3S10Ph intensity was reduced, which may reflect several factors: impaired recruitment of the CPC (containing Aurora B) to chromosomes (possibly secondary to prolonged H3T3 phosphorylation); alterations in chromatin architecture limiting Aurora B’s access to the S10 residue; or indirect inhibition of Aurora B through SEO-induced reduction in CDK levels. Taken together, PP1 and PP2A act on key protein complexes responsible for histone modifications, including the regulation of H3T3 and H3S10 phosphorylation. These modifications are essential for the proper recruitment and activity of the CPC (with Aurora B kinase), which in turn regulates microtubule–kinetochore attachments and spindle checkpoint signaling. Thus, disturbances in histone phosphorylation may impair CPC function, leading to excessive stabilization of microtubules, spindle malfunction, and mitotic delay (Figure 20).

### 3.5. Toxicological Risks Associated with β-Asarone

Essential oils are inherently prone to degradation through oxidation, iso- or polymerization, and dehydrogenation. Due to these properties, they are not considered highly toxic, with acute effects observed only at doses above 2 g/kg (oral or dermal), and their residues on plants remain minimal [15]. Despite the promising phytotoxic activity of SEO, its application as a bioherbicide requires consideration of β-asarone, the main constituent. Mammalian studies have documented its genotoxic and carcinogenic potential, particularly after metabolic activation to β-asarone epoxides, with liver damage and cardiac atrophy observed in rats; the oral LD_50_ is 1010 mg/kg [35]. β- and α-asarone also show toxicity against key agricultural insect pests [119]. Importantly, concentrations of SEO effective for inhibiting plant growth (IC_50_ is 0.05–0.3 g/L) are far below mammalian toxic doses, suggesting that selective application could minimize risks to humans and animals. Nevertheless, field use should account for β-asarone toxicological limits, and further studies are needed on its degradation and environmental residue persistence.

## 4. Materials and Methods

### 4.1. Plant Material

Seeds of faba bean (*Vicia faba* L. subsp. *minor*, cv. Bobas; Danko, HR Sp. z o. o. Choryń, Poland), yellow lupine (*Lupinus luteus* L., cv. Baryt; PHR Sp. z o. o. Tulce, Poland), rapeseed (*Brassica napus* L. cv. Markus; HR Strzelce Sp. z o. o. Strzelce, Poland) and thale cress (*Arabidopsis thaliana* Col-0 (L.) Heynh; NASC, Nottingham, UK.) were germinated on a filter paper in deionized water for 2–3 days at 23 °C in darkness, except *A. thaliana*, which, due to etiolation sensitivity, germinated in long-day conditions (16/8 h). Seedlings with equal-sized primary roots (about 2 cm *V. faba*, *L. luteus*, or 1 cm *B. napus* and 0.5 cm *A. thaliana*) were selected for further experiments.

### 4.2. Essential Oil Treatment

Sweet flag (*A. calamus*) essential oil (SEO) purchased from Etja, Poland, of known composition [97] was emulsified in a 1:4 (*v*/*v*) emulsifier-to-oil ratio by vigorous shaking for 15 min with a mixture of C14-18 and unsaturated C16-18—mono- and di-ethoxylated glycerides and ethoxylated *B. napus* oil. Seedlings were incubated in water-dissolved emulsified SEO, and IC_50_ concentration (the concentration that inhibits root length increment by 50%) was determined for each species. IC_50_ concentration of SEO was: 0.03%—*V. faba*, 0.025%—*L. luteus*, 0.01%—*B. napus*, 0.005%—*A. thaliana*, respectively. Seedlings were incubated on filter paper in tightly sealed Parafilm Petri dishes in darkness for 24 h. The control was seedlings incubated in water and dissolved emulsifier in the concentration used for SEO emulsification. Ten root meristems were examined per species (Control series, emulsifier series—E, and SEO series—O), and four experimental repeats were performed.

### 4.3. DNA Staining for Mitotic and Mitotic Phase Indices

1 cm long apical root fragments were fixed in Carnoy’s solution (99.8% ethanol: glacial acetic acid, 3:1 *v*/*v*) for 1 h, rinsed in absolute ethanol (3 times), and stored in 70% ethanol. Prior to DNA staining, root tips were rehydrated and hydrolyzed in 4 M HCl (for 2 h *V. faba*; 1 h *L. luteus*, *B. napus*; 30 min *A. thaliana*) and stained in Feulgen reaction using Schiff’s reagent (pararosaniline; Sigma-Aldrich, St. Louis, MI, USA) for 1 h [120]. Cut-off meristems were squashed in a drop of 45% acetic acid onto microscopic slides and frozen by placing them on the surface of a dry ice cube. Then, cover slides were removed and the preparations were rinsed in 70% ethanol, air-dried, and mounted in Canada balsam.

### 4.4. Western Blot

Proteins were extracted from the root meristematic parts using Pierce Plant Total Protein Extraction Kit (Thermo Fisher Scientific, Waltham, MA, USA) supplemented with Protease Inhibitor Cocktail (Sigma-Aldrich). Their concentration was spectrophotometrically measured using Pierce BCA Protein Assay Kit (Thermo Fisher Scientific). 45 µg of proteins from each extract were separated on NuPAGE Novex 4–12% Bis–Tris gel (Thermo Fisher Scientific) and immediately transferred onto polyvinylidene fluoride (PVDF) membrane, 0.2 µm pore size (Thermo Fisher Scientific). Membrane blocking, antibody incubation, and chromogenic detection were performed using WesternBreeze Chromogenic Kit, anti-rabbit (Thermo Fisher Scientific) according to the manufacturer’s protocol. Cdc2 proteins were detected using polyclonal Anti-CDC2 antibodies (Agrisera, Vännäs, Sweden) diluted 1:3000, which cross-react with the conserved plant homolog CDKA; therefore, the term ‘Cdc2’ is used throughout this study. Actin was detected using monoclonal actin antibodies (Sigma-Aldrich) diluted 1:4000. The chromogenic reaction was run for 60 and 30 min, respectively. PVDF membranes were visualized using ProXima 2750 imaging platform (Isogen Life Sciences, De Meern, The Netherlands). Microdensitometric analysis of the PVDF membrane was performed using GelAnalyzer 23.1.1 (available at www.gelanalyzer.com (accessed on 1 March 2025) by Istvan Lazar Jr., PhD, and Istvan Lazar Sr., PhD. After loading the membrane image, baselines and bands were automatically detected by the program. Peak intensities were measured by adjusting the band visible on the lane profile and subtracting it from the baseline.

### 4.5. Immunocytochemical Staining of β-Tubulin and Actin

Excised, apical parts of roots were fixed in 4% (m/V) paraformaldehyde dissolved in MTSB buffer (50 mM PIPES, 5 mM EGTA, 5 mM MgSO4, pH 7.0) for 45 min (4 °C) and washed three times with PBS (Sigma-Aldrich). Then root tips were macerated with a citrate-buffered mixture of 2.5% pectinase, 2.5% pectolyase, and 2.5% cellulase (Sigma-Aldrich), at pH 5.0 and 37 °C for 30 min (*V. faba* and *L. luteus*) or 20 min (*B. napus*) or 15 min (*A. thaliana*). After the digestion solution was removed, root tips were washed with PBS as before and squashed onto Super Frost Plus microscope slides (Menzel-Gläser, Thermo Fisher Scientific, Waltham, MA, USA) in a drop of PBS. Following freezing with dry ice, coverslips were removed, and air-dried cells on the slides were permeabilized with PBS-buffered 0.5% Triton X-100 (Sigma-Aldrich) for 15 min, pretreated with blocking buffer (5% BSA, 0.5% Triton X-100 dissolved in PBS) for 50 min (room temperature), briefly rinsed in PBS and incubated with mouse monoclonal anti-β tubulin primary antibodies (Sigma-Aldrich) dissolved in 1:750 ratio in PBS containing 1% w/v BSA and 0.3% *v*/*v* Triton X-100; antibody buffer and in monoclonal actin antibodies (Sigma-Aldrich). Following overnight incubation in darkness and a humidified atmosphere at 4 °C, slides were rinsed three times in PBS and incubated at 23 °C for 90 min with secondary goat anti-mouse antibodies conjugated with Alexa Fluor 488 dissolved in a 1:500 ratio in antibody buffer. After that, the slides were rinsed three times in PBS and stained with 15 µM 4′,6-diamidino-2-phenylindole (DAPI, Sigma-Aldrich) for 15 min. After another rinsing in PBS, the slides were embedded in PBS: glycerol mixture (9:1) with 2.3% DABCO (2.3% diazabicyclo[2.2.2]octane; Sigma-Aldrich).

### 4.6. Immunocytochemical Detection of Phosphorylated H3S10 and H3T3 Histones

Apical fragments of *V. faba* and *B. napus* roots were fixed in 4% (m/V) paraformaldehyde dissolved in PBS buffer for 45 min at 4 °C and washed three times with PBS. Tissue maceration and slide preparation were described above. For H3S10Ph detection, slides were preincubated for 50 min in PBS-buffered 8% BSA and 0.1% Triton X-100 at 23 °C and rinsed twice with PBS buffer for 5 min each. Then, slides were incubated with anti-H3S10Ph antibodies dissolved at 1:300 in PBS containing 1% BSA overnight at 4 °C in darkness in a humidified atmosphere.

To detect H3T3Ph, slides were incubated in permeabilizing solution (0.5% Triton X-100 in PBS) for 15 min at 23 °C, then briefly rinsed and incubated in blocking buffer containing 5% BSA and 0.3% Triton X-100 dissolved in PBS for 50 min at 23 °C. Slides were briefly rinsed and incubated overnight with anti-H3T3Ph antibodies (Cell Signaling Technology, Danvers, MA, USA) dissolved at 1:400 in PBS containing 1% BSA and 0.3% Triton-X100 at 4 °C in darkness in a humidified atmosphere.

After the incubation with primary antibodies stopped, rinsing, incubation in secondary antibodies solution, DAPI staining, and slide mounting procedures were described above.

### 4.7. Microscopic Observations, Measurements, and Analyses

Mitotic indexes and mitotic phase indexes were calculated with the use of Nikon Eclipse E100 microscope (Nikon, Tokyo, Japan) by analyzing 5000 cells from 5 preparations stained with Schiff’s reagent. Calculations were made according to the formula: Mitotic index, MI = (number of dividing cells total number of cells^−1^)∙100%; Phase index, PI = number of cells in a particular phase of mitosis ∙ total number of cells in mitosis^−1^)∙100%

Observations of fluorescence preparations were made using Nikon Eclipse E600W fluorescence microscope (Nikon, Tokyo, Japan) equipped with U2 filter (UVB light; λ = 340–380 nm) for DAPI, B2 filter (blue light; λ = 465–496 nm) for Alexa Fluor^®^ 488, and G2 filter (green light; λ = 540/25 nm) equipped with a Nikon DS-Fil CCD camera (Nikon, Tokyo, Japan) or Zeiss AxioImager. A1 fluorescence microscope (Carl Zeiss, Jena, Germany) equipped with an AxioCam ERc5s CCD camera (Carl Zeiss, Jena, Germany). All images were recorded at the same exposure time. Quantitative analyses of fluorescence intensity were made in ImageJ software ver. 1.54p [121] after converting color images into greyscale and expressed in arbitrary units.

### 4.8. Statistical Analyses

Statistical analyses and outcomes visualization were performed in GraphPad Prism 10 software. Differences between groups were evaluated using Student’s *t*-test or the Mann–Whitney test. Statistical significance, denoted by asterisks (*) or hash symbols (#), was attributed to *p*-values ≤ 0.05.

## 5. Conclusions

The results indicate that SEO reduces the proportion of mitotically active cells and disrupts mitotic progression in representatives of both Fabaceae (*V. faba*, *L. luteus*) and Brassicaceae (*B. napus*, *A. thaliana*). Application of species-specific IC_50_ concentrations revealed the universality of SEO’s main effects while also demonstrating species-specific differences in response intensity.

The action of SEO proved to be multifaceted, encompassing molecular mechanisms of cell cycle regulation, dynamic remodeling of cytoskeletal elements (microtubules and actin filaments), and mitosis-specific histone modifications such as H3T3 and H3S10 phosphorylation. These epigenetic alterations exhibited interspecific differences in both intensity and spatial distribution.

The reduction in the number of mitotic cells results from both replication stress and S-phase prolongation, caused by oxidative DNA damage, as confirmed in earlier studies, and from a decrease in the level of the key cell cycle regulatory kinase Cdc2 (a protein that normally remains relatively stable throughout the cell cycle), as demonstrated in *V. faba* and *B. napus* by Western blotting.

Mitotic disturbances were also associated with excessive stabilization of microtubules and actin filaments, and with restricted dynamic reorganization of the spindle and phragmoplast, observed in all studied species by immunofluorescence as stronger lateral interactions and persistent stabilization at each mitotic stage. It is likely that microtubule-associated proteins (MAPs) mediate these lateral interactions; under reduced Cdc2 levels, they may fail to undergo phosphorylation required for weakening their microtubule binding and enabling proper separation.

Mitotic defects were further linked to altered timing and intensity of H3T3 phosphorylation, which is essential for correcting erroneous kinetochore–microtubule attachments and for proper spindle organization, as shown in *V. faba* and *B. napus* by immunofluorescence. This disruption is likely due to SEO interference with PP1/2A phosphatases, responsible both for H3 dephosphorylation and for Haspin kinase inactivation, although this assumption requires further validation.

Disturbances in H3T3 phosphorylation timing may also have hindered CPC/Aurora B recruitment to chromatin, thereby weakening H3S10 phosphorylation, as demonstrated in *V. faba* and *B. napus*. In addition, the observed decrease in Cdc2 levels could further reduce Aurora B activity, limiting its ability to phosphorylate H3S10 and to ensure proper chromatin condensation into metaphase chromosomes and accurate sister chromatid separation. This mechanism, however, requires further confirmation.

From the perspective of the potential use of SEO as a bioherbicide, it is particularly important to emphasize its capacity to target multiple components of the cell cycle. This multi-targeted mode of action may reduce the risk of plants rapidly developing resistance mechanisms, which is a key advantage of such compounds in agricultural practice.

## Data Availability

Data will be made available upon request.

## Abbreviations

The following abbreviations are used in this manuscript:

APC/C	Anaphase-promoting complex/cyclosome
ATM	Ataxia-telangiectasia mutated kinase
ATR	Ataxia-telangiectasia and Rad3-related protein
BMF3	BUB1/MAD3 family protein 3
CAK	Cdk-activating kinase
Cdc2	Cell division control 2
CDC20	Cell division cycle 20 protein
CDC25	Cell division cycle 25 phosphatase
CDKs	Cyclin-dependent kinases
CKIs	Cyclin-dependent kinase inhibitors
CPC	Chromosomal passenger complex
Cyc	Cyclin
DP	Dimerization partner
MAD2	Mitotic arrest deficient 2
MAPs	Microtubule-associated proteins
MAPK	Mitogen-activated protein kinase
MCC	Mitotic checkpoint complex
MPF	Mitosis-promoting factor
NACK1	NPK1-activating kinesin-like protein 1
PP1-2A, B, C	Protein Phosphatase 1-2A, B, C
PPB	Preprophase band
Rb	Retinoblastoma protein
RBR	Retinoblastoma-related proteins
ROS	Reactive oxygen species
SAC	Spindle assembly checkpoint
SMC	Structural maintenance of chromosome protein
TPX2	Targeting Protein of XKLP2
γ-TuRC	γ-tubulin ring complex
